# Human gastric multi-regional assembloids for functional parietal maturation and patient-specific modelling of antral foveolar hyperplasia

**DOI:** 10.1038/s41551-025-01553-y

**Published:** 2026-01-23

**Authors:** Brendan C. Jones, Giada Benedetti, Giuseppe Calà, Ramin Amiri, Lucinda Tullie, Roberto Lutman, Jahangir Sufi, Lucy Holland, Daniyal J. Jafree, Monika Balys, Glenn Anderson, Ian C. Simcock, Owen J. Arthurs, Simon Eaton, Nicola Elvassore, Vivian SW Li, Christopher J. Tape, Kelsey DJ Jones, Camilla Luni, Giovanni Giuseppe Giobbe, Paolo De Coppi

**Affiliations:** 1 Stem Cell and Regenerative Medicine Section, Great Ormond Street Institute of Child Health, University College London, London, UK; 2 Department of Surgical Biotechnology, Division of Surgery and Interventional Science, University College London, London, UK; 3 Department of Civil, Chemical, Environmental and Materials Engineering, University of Bologna, Bologna, Italy; 4 Stem Cell and Cancer Biology Lab, The Francis Crick Institute, London, UK; 5 National Institute for Health and Care Research Great Ormond Street Hospital Biomedical Research Centre, London, UK; 6 Cell Communication Lab, Department of Oncology, University College London Cancer Institute, London, UK; 7 Developmental Biology and Cancer Research and Teaching Department, Great Ormond Street Institute of Child Health, University College London, London, UK; 8 Wellcome Genome Campus, Wellcome Sanger Institute, Hinxton, UK; 9 Department of Histopathology, Great Ormond Street Hospital, London, UK; 10 Department of Radiology, Great Ormond Street Hospital, London, UK; 11 Population, Policy and Practice Research and Teaching Department, Great Ormond Street Institute of Child Health, University College London, London, UK; 12 Veneto Institute of Molecular Medicine, Padua, Italy; 13 Department of Biomedical Sciences, University of Padua, Padua, Italy; 14 Department of Paediatric Gastroenterology and Nutrition, Great Ormond Street Hospital, London, UK; 15 Department of Specialist Neonatal and Paediatric Surgery, Great Ormond Street Hospital, London, UK

**Keywords:** Biomedical engineering, Gastrointestinal models, Adult stem cells

## Abstract

Patient-derived human organoids have the capacity to self-organize into more complex structures. However, to what extent gastric organoids can recapitulate differentiated cell types and mucosal functions remains unexplored. Here we report on how region-specific gastric organoids can self-assemble into complex multi-regional assembloids. These assembloids show increased complexity and cross-communication between different gastric regions, allowing for the emergence of the elusive parietal cell type that is responsible for the production of gastric acid and shows a functional response to drugs targeting the H^+^/K^+^ ATPase pump. We generate assembloids from paediatric patients with a genetic condition found to be associated with unusual antral foveolar hyperplasia and hyperplastic polyposis. Our multi-regional assembloid efficiently recapitulates hyperplastic-like antral regions, with decreased mucin secretion and glycosylated H^+^/K^+^ ATPase subunit beta, which results in impaired gastric acid secretion. Multi-regional gastric assembloids, generated using paediatric-stem-cell-derived organoids, successfully recapitulate the structural and functional characteristics of the human stomach, offering a promising tool for studying gastric epithelial interactions and disease mechanisms that were previously challenging to investigate in primary models.

## Abstract (web summary)

In vitro models using tissue-specific progenitors recapitulate the lumen with gland-like structures and functional characteristics of the human stomach epithelium in a patient-specific disease model.

## Main

The human stomach is a vital organ responsible for the initial phases of food digestion and relies on finely regulated hormonal signalling to function. As a complex and dynamic system, the stomach plays a crucial role in the overall digestive process, enabling the breakdown of ingested food into absorbable nutrients. Structurally, it comprises three main distinct regions—fundus, body and antrum—each with contrasting morphological and functional properties^1,2^. Two types of gastric glands with distinct locations have been described: oxyntic and antral glands. The oxyntic glands, located in the fundus and body, are characterized by an abundance of parietal cells and mucin-secreting cells in the neck, and are essential for the secretion of hydrochloric acid and intrinsic factor. The antral glands have a longer pit domain with neck mucous and endocrine cells (predominantly G cells at the base) and are responsible for producing the gastrin hormone, which plays a central role in regulating gastric acid secretion in the fundus^2,3^.

During human development, the antrum forms prior to the fundus, usually around the fourth week of gestation. The antrum develops from the caudal portion of the primary stomach, and later the fundus develops from the cranial portion^4^, with regional specification occurring in the early stages of embryonic formation. Animal models are not suited to the investigation of human gastric cross-regional interactions (that is, the mouse stomach, in contrast with the human one, comprises an antrum, corpus and forestomach, with the forestomach presenting a keratinizing stratified epithelium)^5^. In the past decade, primary gastric organoid models, which comprise epithelial cells only, have emerged as a useful tool for modelling various physiological and pathological processes in the stomach, thanks to their ability to recapitulate some gastric functions and retain genetic diversity and patient-specific features^6–9^. Alternatively, gastric organoids derived from human pluripotent stem cells (hPSC) are advantageous as they also contain mesenchyme. However, in contrast to primary organoids, achieving regionalization in hPSC-derived organoids is more complex and time consuming using current protocols^10^.

Although primary organoids provide valuable insights into gastric biology, they are still relatively simple models compared with native tissue, lacking features such as cellular complexity and function that are challenging to reproduce in vitro. Similar limitations were observed in organoids derived from different intestinal regions, which may recapitulate some of the biological characteristics of the specific region, but fail to fully reflect the complexity of epithelial function^9,11^.

One of the major limitations of primary gastric models is related to the absence of more complex cell interactions, which render simple organoids an incomplete tool for modelling the stomach^11^. For instance, functional acid-producing parietal cells, present in gastric tissue biopsies, are poorly represented in murine primary gastric epithelial organoids^12^. Still, examples of murine-derived fundic organoids presenting parietal cells have been reported upon addition of mesenchymal components^13^. Furthermore, the possibility to induce the differentiation of parietal cells has been demonstrated with hPSC-derived fundic gastric organoids^14^. However, the appearance of functional parietal cells has never been reported in human tissue-derived gastric organoids^7,15^; it has only been reported in primary monolayer models upon alteration of media composition^16^.

Investigating the dynamic interplay between the antrum, body and fundus is essential for enhancing our understanding of the mechanisms that regulate gastric physiological functions, as well as associated pathologies. For example, disorders related to disrupted acid production within the stomach may be caused by dysregulation of the different regions of the stomach during development or later in life^17^. Gastric-acid-related diseases constitute a spectrum of gastrointestinal conditions that arise from disruptions in stomach functions, exerting profound effects beyond the gastrointestinal tract. This encompasses ailments such as gastritis, peptic ulcers and gastroesophageal reflux disease, often stemming from aberrant gastric acid production^17^. To date, treatments have focused on symptoms management and the prevention of long-term complications. However, little has been done to investigate the dysregulated pathology leading to gastric disorders.

To overcome these challenges and advance our understanding of the gastric interactions between the antrum and fundus, additional research approaches are desired. In this context, the development of a gastric assembloid model emerges as a promising strategy^18^. The gastric multi-regional assembloids (MRAs) we propose here are advanced in vitro models that recapitulate the structural (lumen and gland-like structures) and functional (mucin and acid secretion) characteristics of the human stomach epithelium using patient-derived tissue-specific progenitors.

To assess the applicability of our system for disease modelling, we generated primary gastric epithelial cell organoids from patients with phosphomannomutase 2-associated hyperinsulinism with polycystic kidney disease and inflammatory bowel disease (PMM2–HIPKD–IBD). *PMM2* encodes phosphomannomutase 2, which is a non-redundant component in the N-glycosylation pathway. Patients with biallelic deleterious mutations in *PMM2* suffer from a congenital disorder of glycosylation that is associated with complex multisystemic manifestations incorporating pervasive neurological features. In contrast, patients with PMM2–HIPKD–IBD harbour a monoallelic deleterious mutation in *PMM2* in *trans* with a specific *PMM2* promotor mutation, or the promotor mutation in homozygosity, and have a more restricted pattern of disease incorporating hyperinsulinism, polycystic kidney disease (PKD) and inflammatory bowel disease (IBD). These patients also appear to have a tendency towards the development of gastric antral foveolar hyperplasia and antral hyperplastic polyposis in early childhood^19^. Such gastric manifestations are very unusual in children, and have not been identified in association with any other monogenic IBD syndrome to our knowledge. The cellular and molecular mechanism(s) underlying this tendency are unknown, but in general the organ-level pattern of disease in PMM2–HIPKD–IBD reflects the tissue-specific expression of the transcription factor encoded by the gene *HNF4A*. It has been hypothesized that the promotor mutation interrupts HNF4α *cis*-acting regulatory control of *PMM2* expression, resulting in critically reduced PMM2 activity in a restricted set of cells and tissues^19^. Based on published expression data, it was proposed that the gastroenterological manifestations of PMM2–HIPKD–IBD probably represent an epithelial impairment^19^. Exploiting the potential of the gastric MRAs, we were able to model some features of gastric antral foveolar hyperplasia in vitro.

We have demonstrated that the organoids retain native regional specificity after isolation from gastric tissue. When combined to generate MRAs, we can generate an in vitro system that more closely recapitulates the stomach epithelium. Indeed, we observed the spontaneous emergence of functional parietal cells that were absent in other epithelial organoid systems in expansion medium. MRA models may enable controlled manipulation, as well as the observation of specific cellular components and signalling pathways, providing insights into spatial interactions, and disease mechanisms that would otherwise be unattainable.

## Results

### Gastric organoids from the fundus, body and antrum retain regional memory

**Fig. 1:**
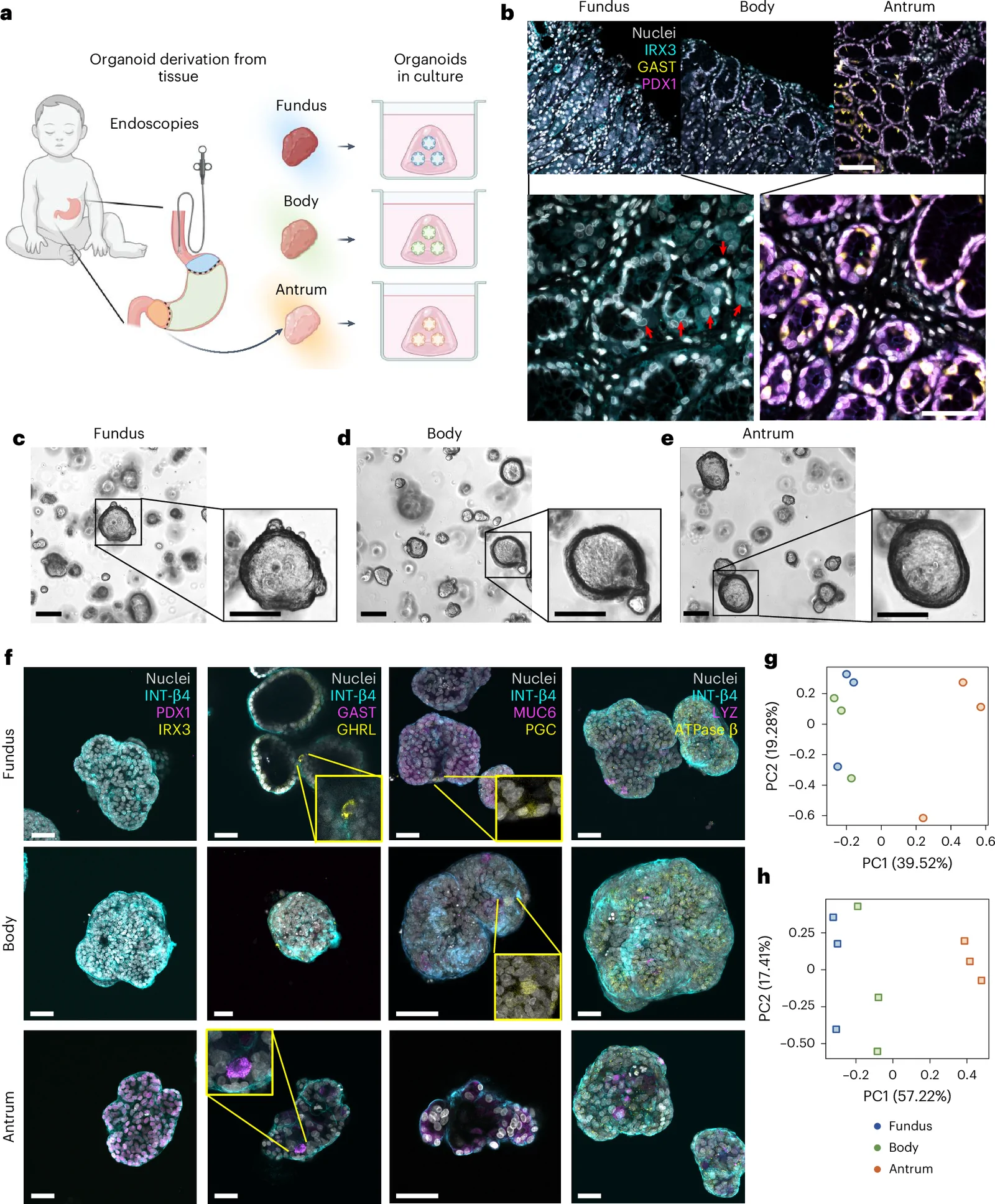
Characterization of gastric regionality in tissues and organoids. **a**, Schematic of organoid derivation from gastric mucosal biopsies from paediatric patients. **b**, Immunofluorescence panel showing fundus, body and antrum paediatric stomach tissue sections stained for IRX3 (cyan), GAST (yellow), PDX1 (magenta) and nuclei (grey; Hoechst). Scale bars, 100 μm (top) and 50 μm (bottom). Red arrows indicate IRX3-positive nuclei within the glands. **c**–**e**, Brightfield images of a representative organoid line for the fundus (**c**), body (**d**) and antrum (**e**) regions, showing the formation of spherical organoids within 7 days, starting from single cells. Scale bars, 200 μm. **f**, Immunofluorescence panel showing integrin β4 (INT-β4; cyan); PDX1, GAST, MUC6 and LYZ (magenta); IRX3, GHRL, PGC and H^+^/K^+^ ATPase subunit β (yellow); and nuclei (grey; Hoechst) in fundus, body and antrum organoids. Scale bars, 20 μm. **g**,**h**, PCA of RNA-seq samples from fundus (blue), body (green) and antrum (red) tissue (**g**) and organoids (**h**) (*n* = 3; patient 1). Panel **a** created with BioRender.com. Source Data

**Extended Data Fig. 1:**
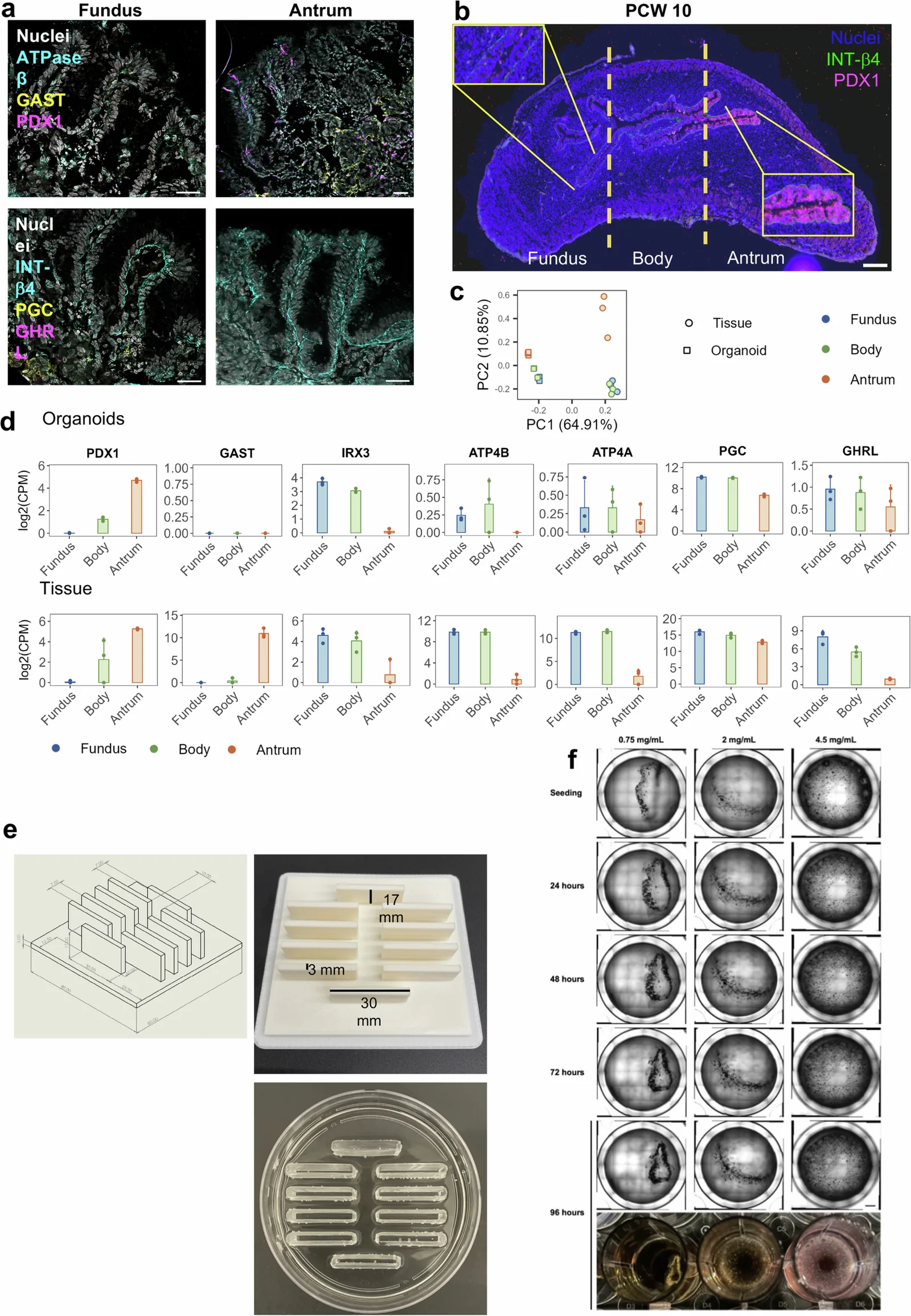
Tissue regionality and organoid characterization and assembloid protocol generation set-up. **a**, Immunofluorescence panel showing Fundus and Antrum in paediatric stomach tissue sections stained for ATPase H^+^/K^+^ transporting subunit β (ATPase β) in cyan, Gastrin (GAST) in yellow, Pancreatic and Duodenal homeobox 1 (PDX1) in magenta, and nuclei in grey (Hoechst); or Integrin-β4 (INT-β4) in cyan, Pepsinogen C (PGC) in yellow, Ghrelin (GHRL) in magenta, and nuclei in grey (Hoechst). Scale bar 25 μm. **b**, Immunofluorescence panel showing foetal stomach (post-conception week 10) tissue sections stained for Integrin-β4 (INT-β4) in green, Pancreatic and Duodenal homeobox 1 (PDX1) in magenta, and nuclei in blue (Hoechst). Scale bar 250 μm. **c**, PCA of RNA-sequencing samples from Fundus (blue), Body (green), and Antrum (red) tissue (circles) versus organoids (squares). (n = 3, patient 1). **d**, Expression of typical gastric markers in tissue and organoids in Fundus (blue), Body (green), and Antrum (red). Circles indicate single data points, error bar indicates mean ±SD (n = 3, patient 1). CPM: counts per million. **e**, Custom-designed well for assembloid seeding: mould design (left), 3D printed mould (top right), PDMS customized-well (bottom right). **f**, SRA formation at various concentrations of collagen I hydrogel (0.75, 2, or 4.5 mg/ml). Scale bar 2 mm. Source Data

To determine whether gastric stem cells can retain the identity of the region of origin, we isolated mucosal biopsies from the antrum, body and fundus regions of four paediatric patients (Fig. 1a and Supplementary Table 1). We verified the presence of the regional markers in paediatric tissues. Antral tissue showed the presence of gastrin (G cells) and pancreatic and duodenal homeobox protein 1 (PDX1), with PDX1 absent in the fundus and body epithelium, whereas fundus tissue showed defined iroquois homeobox 3 (IRX3) marker expression (Fig. 1b and Extended Data Fig. 1a). This is in keeping with PDX1 expression in post-conception-week-10 human stomach, which is limited to the antral cells, showing the early establishment of gastric regionality (Extended Data Fig. 1b)^20,21^.

Gastric organoids were derived according to previously established protocols^8,22^ and cultured in gastric-specific medium^23^. The organoid cultures generated from stomach fundus, body and antrum showed a comparable cystic morphology (Fig. 1c–e). Fully grown organoids (day 7 of culture) were immunostained for regional and gastric-specific markers. Strikingly, PDX1 specifically marked antrum-derived organoids only, in which we detected a low proportion of cells positive for gastrin (GAST), mucin 6 (MUC6) and lysozyme (LYZ). Fundus and body organoids exhibited cells positive for LYZ, MUC6, ghrelin (GHRL) and pepsinogen C (PGC). Importantly, H^+^/K^+^ ATPase subunit beta (ATPase β) was not detected in any of the organoids (Fig. 1f).

We then characterized native tissues (mucosal controls) and regional organoids with bulk RNA sequencing (RNA-seq). Overall, principal component analysis (PCA) on the tissue showed the antral samples separating from the cluster, including fundus and body samples (Fig. 1g). The same trend was found when analysing organoid samples, with antral organoids clustering separately from the other two regions (Fig. 1h). When comparing tissue and organoids, we could appreciate the presence of a compact cluster defined by the organoids of all regions. While strongly diverging from the organoid cluster, the tissue samples from the three regions were more scattered, with the antrum separating from the other two regions (Extended Data Fig. 1c). Looking at specific regionality targets, *PDX1* was expressed by the antrum in both tissue and organoids. *GAST* (expressed by G cells in the antrum), *ATP4A* and *ATP4B* (expressed by parietal cells in the body and fundus) and *GHRL* (expressed in P/D1 or X cells in the body and fundus) were respectively found to be expressed in tissue, but almost absent in organoids. Transcripts of *IRX3* (a transcription-factor-encoding gene mainly expressed in the body and fundus) and *PGC* (expressed in chief cells, more strongly in the body and fundus) were present in tissue, and more sparsely in organoids (Extended Data Fig. 1d).

### Gastric organoids self-organize to form complex assembloids

Mature cell types in primary gastric organoids were scarcely detected, or absent (for example, parietal cells, G cells and X/A endocrine cells), suggesting that organoid expansion alone is not sufficient to recapitulate full epithelial gastric function. To date, parietal cells have been observed transiently in hPSC-derived gastric organoids^10,14^, and primary air–liquid interface cultures in a differentiation medium^16^. However, spontaneously emerging parietal cells have never been reported in human primary organoids in an expansion medium^7^. The literature has reported an overall difficulty in their identification in primary murine models as well^12^, but there are examples of murine-derived fundic organoids presenting parietal cells^13^.

**Fig. 2:**
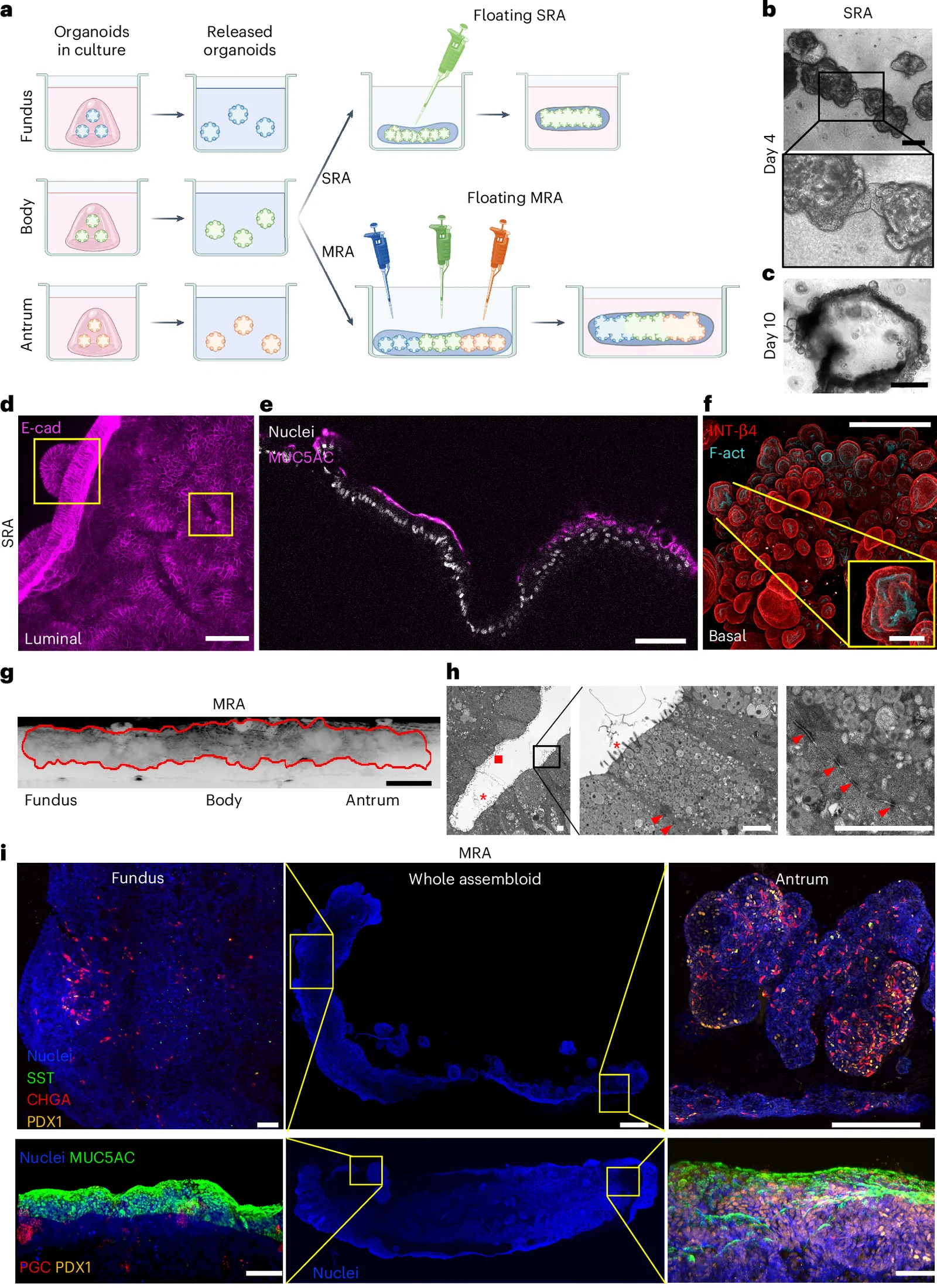
Gastric assembloid generation and characterization. **a**, Schematic of SRA and MRA generation. **b**, Brightfield image of organoid fusion at day 4 of floating collagen I hydrogel culture. Scale bar, 200 μm. **c**, Brightfield image of an SRA at 10 days. Scale bar, 1 mm. **d**, Whole-mount imaging of a gastric SRA from the luminal side, showing E-cadherin (E-cad) in magenta. The yellow squares highlight an aperture in the lumen and a bud-like protrusion emerging from the lumen. Scale bar, 100 μm. **e**, SRA section stained for nuclei (white; RedDot) and MUC5AC (magenta). Scale bar, 50 μm. **f**, Whole-mount images of gastric SRAs, showing INT-β4 (red) and F-actin (F-act; cyan). The image was processed with Imaris. Scale bars, 500 μm (main image) and 100 μm (inset). **g**, Brightfield image of an MRA at 10 days. Scale bar, 1 mm. **h**, TEM images of MRAs. The grey circles within the cells indicate mucin-filled granules. The square, asterisks and arrowheads represent a gland, microvilli and desmosomes, respectively. Scale bars, 2 μm. **i**, Whole-mount immunofluorescence image of a gastric MRA, showing nuclei (blue; Hoechst), somatostatin (SST; green), CHGA (red) and PDX1 (orange) or nuclei (blue; Hoechst), MUC5AC (green), PGC (red) and PDX1 (orange). The central column provides an overview of the whole tube structure (scale bar, 500 μm) whereas the left and right columns show high magnification (scale bars, 50 μm). Panel **a** created with BioRender.com.

Therefore, we designed a system to allow the self-aggregation of human gastric organoids in more complex multi-regional structures, defined as assembloids (that is, MRAs), where cells can reach maturity and functionality (Fig. 2a). To facilitate organoid self-assembly while maintaining the regional and orientational information, we designed a customized polydimethylsiloxane (PDMS)-based culture well (Extended Data Fig. 1e). Briefly, fully grown organoids were released from Matrigel, resuspended in collagen I hydrogel and cultured under floating conditions, after assessment of the ideal gel concentration at 0.75 mg ml^−1^ (Extended Data Fig. 1f and Supplementary Video 1).

**Extended Data Fig. 2:**
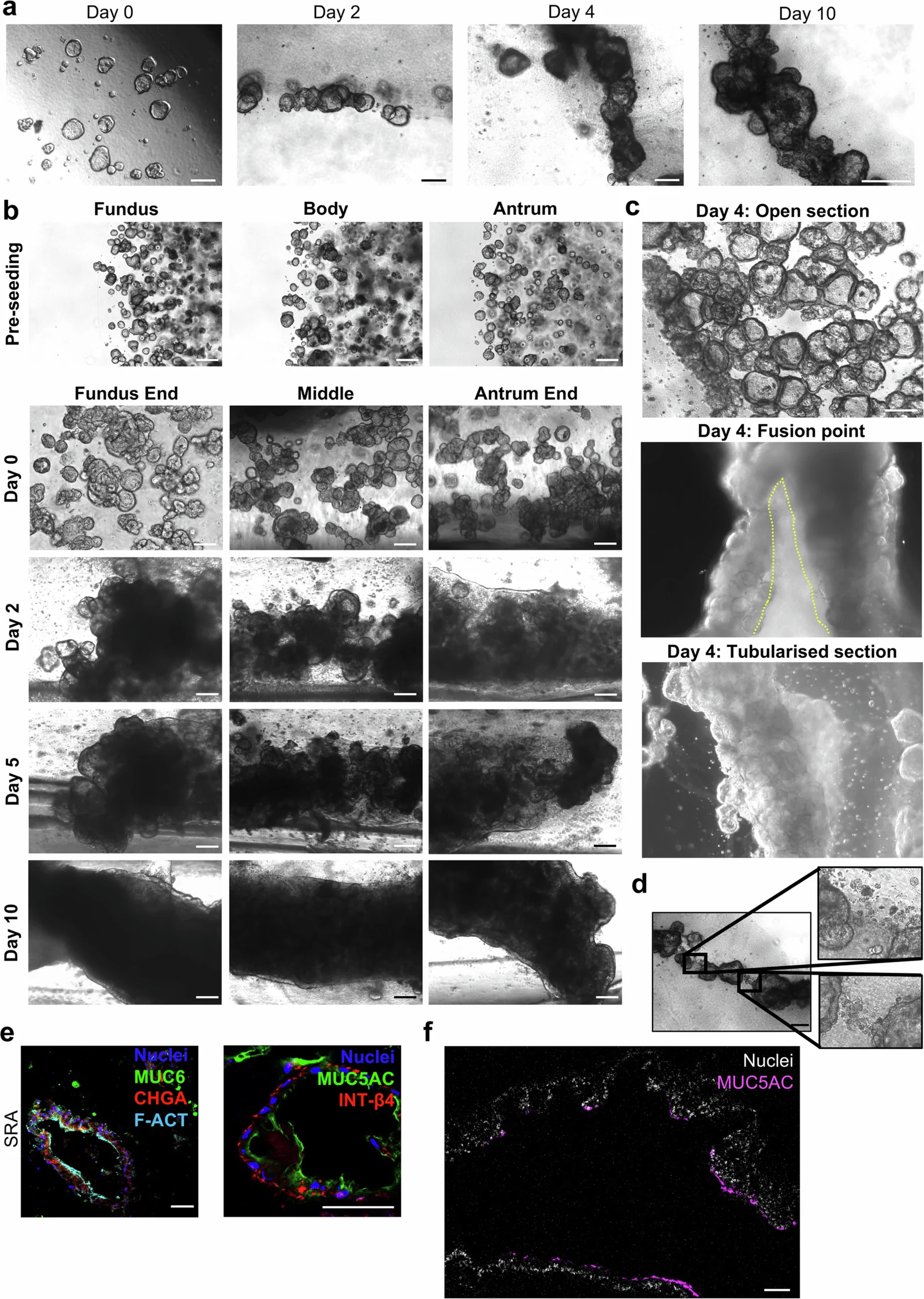
Assembloid generation. **a**, Morphology of SRA formation when seeded at a density of 6000 organoids per mL over 10 days in floating collagen I hydrogel culture. Scale bars, 200 μm. **b**, Bright field images of MRA formation at Fundus, Body, and Antrum over 10 days in floating collagen I hydrogel culture. Scale bars, 200 μm. **c**, Bright field images of different parts of SRA at day 4 in culture: open section (upper panel), V-shaped fusion point marked by dotted yellow line (middle panel), tabularised section (lower panel). Scale bar, 200 μm. **d**, Bright field images of different parts of SRA at day 4 in culture showing syncytium formation. Scale bar, 200 μm. **e**, Immunofluorescence of sectioned gastric SRA showing mucin 6 (MUC6) in green, chromogranin A (CHGA) in red, F-actin (F-ACT) in cyan, and nuclei in blue (Hoechst); or mucin 5AC (MUC5AC) in green, integrin β4 (INT-β4) in red, and nuclei in blue (Hoechst). Scale bars, 50 μm. **f**, SRA section stained for Nuclei in white (RedDot), and Mucin 5AC (MUC5AC) in magenta (scale bar 50 μm).

We first defined the self-assembly conditions for each region (hereafter noted as single-region assembloids (SRAs)) and then MRAs assembled in the order fundus–body–antrum, to discern whether merely increasing the structural complexity facilitates increased functionality, or whether multi-regionality is needed to enhance this further. The related organoid/collagen structures were cultured in suspension for 10 days, allowing contraction and self-aggregation (Extended Data Fig. 2a,b). By day 4, fusion between organoids could be observed (Fig. 2b and Extended Data Fig. 2c,d). By day 10, the self-assembled SRA and MRA structures were fully formed and used for subsequent analysis (Fig. 2c,g). SRAs showed a layer of epithelial cells polarized towards a lumen, and the expression of mucin 5AC (MUC5AC), MUC6 and chromogranin A (CHGA) confirmed SRA gastric identity (Extended Data Fig. 2e). Whole-mount staining on SRAs showed the presence of a continuous shared lumen, with luminal apertures connecting to protrusions from the main assembloid body (Fig. 2d). SRA transverse sections showed mucin secretion in the luminal domain and absence in the budding domains (Fig. 2e and Extended Data Fig. 2f). These gastric epithelial gland-like structures were characterized by apicobasal cell polarity determined by luminal F-actin and basal integrin β4 (Fig. 2f).

We performed transmission electron microscopy (TEM) analysis on MRAs and identified gastric gland-like structures with a mature epithelial monolayer of mucus-secreting cells. We confirmed the presence of microvilli on the apical domain, as well as desmosomes mediating cell adhesion on the lateral domains (Fig. 2h). Remarkably, immunofluorescence analysis on MRAs demonstrated the preservation of regional identity within the assembloid system. PDX1 was found at the antral end of MRAs, together with enterochromaffin-like cells (chromogranin A) and somatostatin-positive cells. Additionally, MRAs revealed high expression of MUC5AC, indicating the presence of active mucin-producing cells, and PGC^+^ cells were exclusively identified at the fundic end (Fig. 2h and Supplementary Videos 2 and 3).

### Cross-talk pathways arise in gastric MRAs

**Fig. 3:**
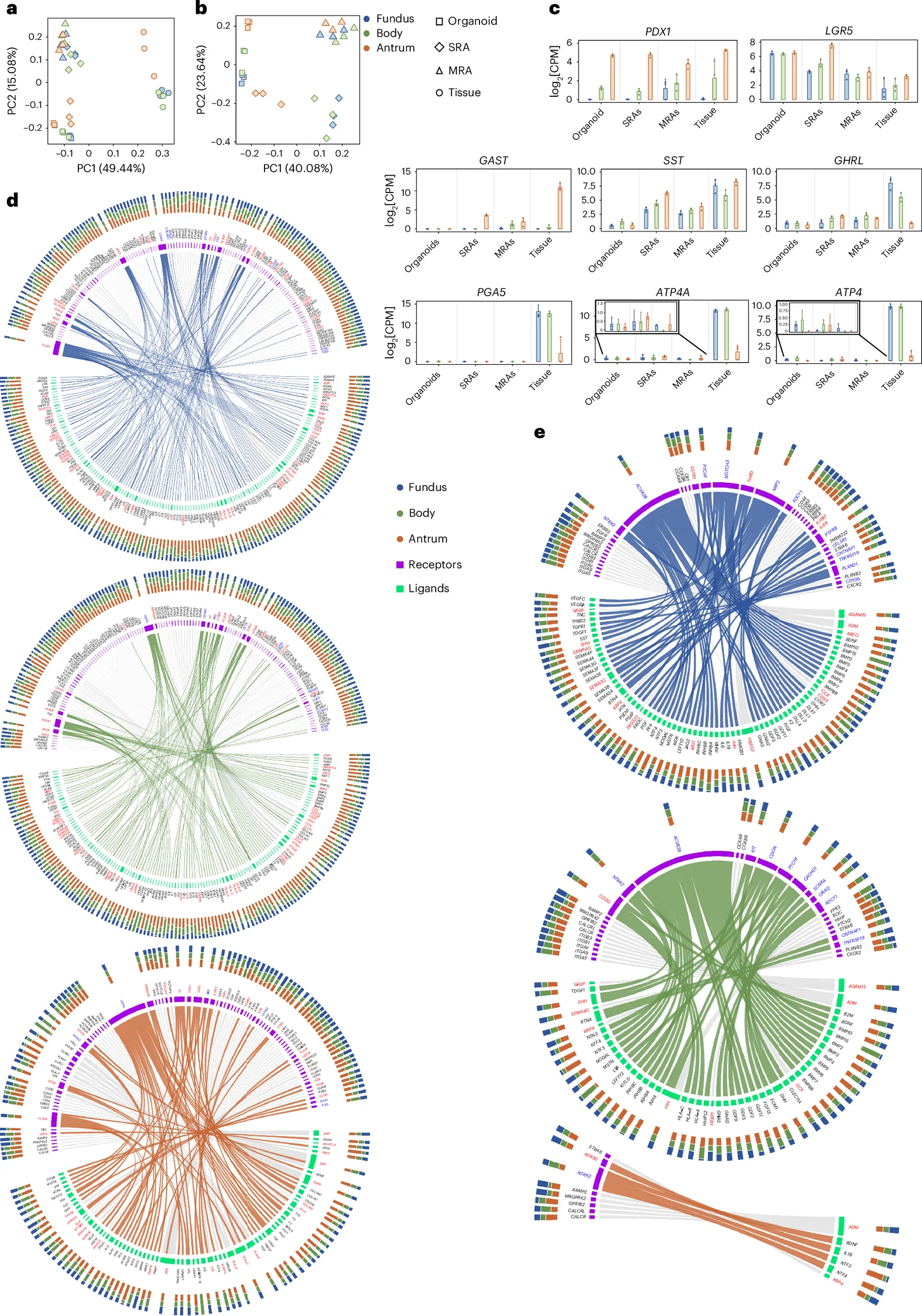
Gastric assembloid bulk RNA-seq analysis. **a**,**b**, PCA of RNA-seq samples from fundus (blue), body (green) and antrum (red) organoids (squares), SRAs (rhomboids) and MRAs (triangles). Panel **a** also includes the results for tissue (circles) (*n* = 3 for both; patient 1). **c**, Expression of typical gastric markers in organoids, SRAs, MRAs and tissue in the fundus (blue), body (green) and antrum (red). The circles indicate single data points and the error bars represent means ± s.d. (*n* = 3; patient 1). **d**,**e**, Chord diagrams displaying ligand–receptor analysis in organoids versus MRAs (**d**) and in SRAs versus MRAs (**e**). The fundus, body and antrum are represented in blue, green and orange, respectively. Receptors are noted with a purple bar and ligands with a green one. Upregulated genes are reported in red, downregulated genes are in blue and non-differentially expressed genes are in black (|fold change| > 1.5; false discovery rate (FDR) < 0.05). The external bar plots show the proportion of expression in the antrum, body and fundus of MRAs for every gene. For each of the ligand–receptor couples considered, at least one of the two genes of the couple was differentially expressed (only upregulated) across the four patients analysed at |fold change| > 2 and FDR < 0.01 (*n* = 9; patient 1 in triplicate; patients 2, 3 and 4 in duplicate). Source Data

**Extended Data Fig. 3:**
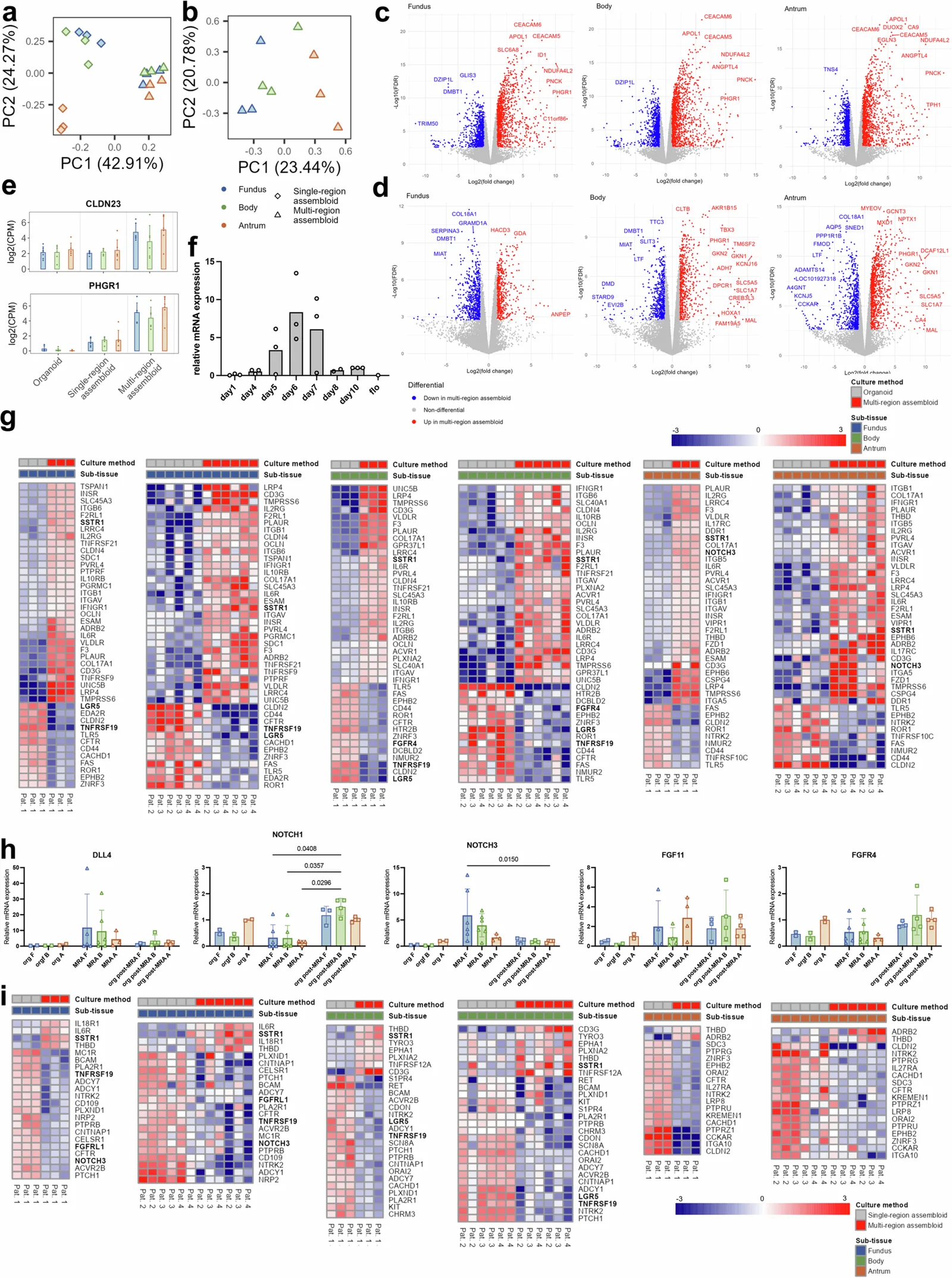
Transcriptomics analysis. **a**, Principal component analysis (PCA) of RNA-sequencing samples from Fundus (blue), Body (green), and Antrum (red) SRA (rhomboids) versus MRA (triangles). (n = 3, patient 1). **b**, Principal component analysis (PCA) of RNA-sequencing samples from Fundus (blue), Body (green), and Antrum (red) MRA (triangles). (n = 3, patient 1). **c**, Volcano plots of DEGs between organoids and MRA of Fundus, Body, and Antrum regions (n = 3, patient 1). **d**, Volcano plots of DEGs between SRA and MRA of Fundus, Body, and Antrum regions. (n = 3, patient 1). **e**, Expression of CLDN23 and PHGR1 in organoids, SRA and MRA in Fundus (blue), Body (green), and Antrum (red). Circles indicate single data points, mean ±SD (n = 6, patient 2,3,4) CPM: counts per million. **f**, RT-qPCR for *ATP4B*whole MRA at progressive days during MRA formation, normalized to day 10. Mean and single datapoints displayed (n = 3). FLO (foetal lung organoids) were used as a negative control. **g**, Hierarchical clustering of receptors that are differentially expressed genes (DEGs) between organoids and MRA in the three regions intersected among 4 patients. Color bar indicates centered log2(CPM + 1). Patient 1: |fold change | >2, FDR < 0.01), patients 2, 3, and 4: |fold change | >1.5, FDR < 0.05). **h**, RT-qPCR for ligands and receptors in organoids (n = 2), MRA (n = 6), and organoids grown from MRA (n = 4) as labelled (patient 1). Single datapoints displayed, mean±SD. One way ANOVA **i**, Hierarchical clustering of receptors that are differentially expressed genes (DEGs) between SRA and MRA in the three regions intersected among 4 patients. Colorbar indicates centered log2(CPM + 1). Patient 1: |fold change | >2, FDR < 0.01), patients 2, 3, and 4: |fold change | >1.5, FDR < 0.05). Source Data

Following RNA-seq analysis, PCA of fundus, body and antrum from tissue, organoids, SRAs and MRAs showed that all of the in vitro systems clustered separately from the native tissue samples (Fig. 3a). A second analysis excluding tissue samples and considering only in vitro systems showed how each sub-group clusters separately (Fig. 3b). In addition, a separate PCA conducted on SRAs and MRAs only, indicated how these two groups cluster separately. Also, the antrum showed a clear separation from the body and fundus in both SRAs and MRAs (Extended Data Fig. 3a,b). Overall, MRAs displayed a variety of differentially expressed genes (DEGs), such as the epithelial maturation markers *CLDN23* and *PHGR1* (ref. ^24^), compared with both organoids (Extended Data Fig. 3c,e) and SRAs (Extended Data Fig. 3d,e), suggesting an advantage in the multi-regional complex system^25^.

Looking at selected gastric targets, *PDX1* was confirmed to be antral specific in all sample types. The stem cell marker *LGR5* showed a reduction in MRAs compared with other in vitro systems. Endocrine cell markers (*GAST*, *SST* and *GHRL*) were found to be present in the assembloids in coherence with their regional expression in the tissue. Parietal cell marker (*ATP4A* and *ATP4B*) messenger RNAs (mRNAs) were lowly expressed in all in vitro systems compared with gastric tissues (Fig. 3c), although RNA expression was still present (magnified insets in Fig. 3c). Of note, daily quantitative PCR with reverse transcription (RT-qPCR) during the 10-day MRA formation showed how the *ATP4B* mRNA peaked at around day 6, then decreased by day 8 (Extended Data Fig. 3f). Therefore, this temporal dynamic is coherent with the low mRNA detection at the experimental endpoint.

**Extended Data Fig. 4:**
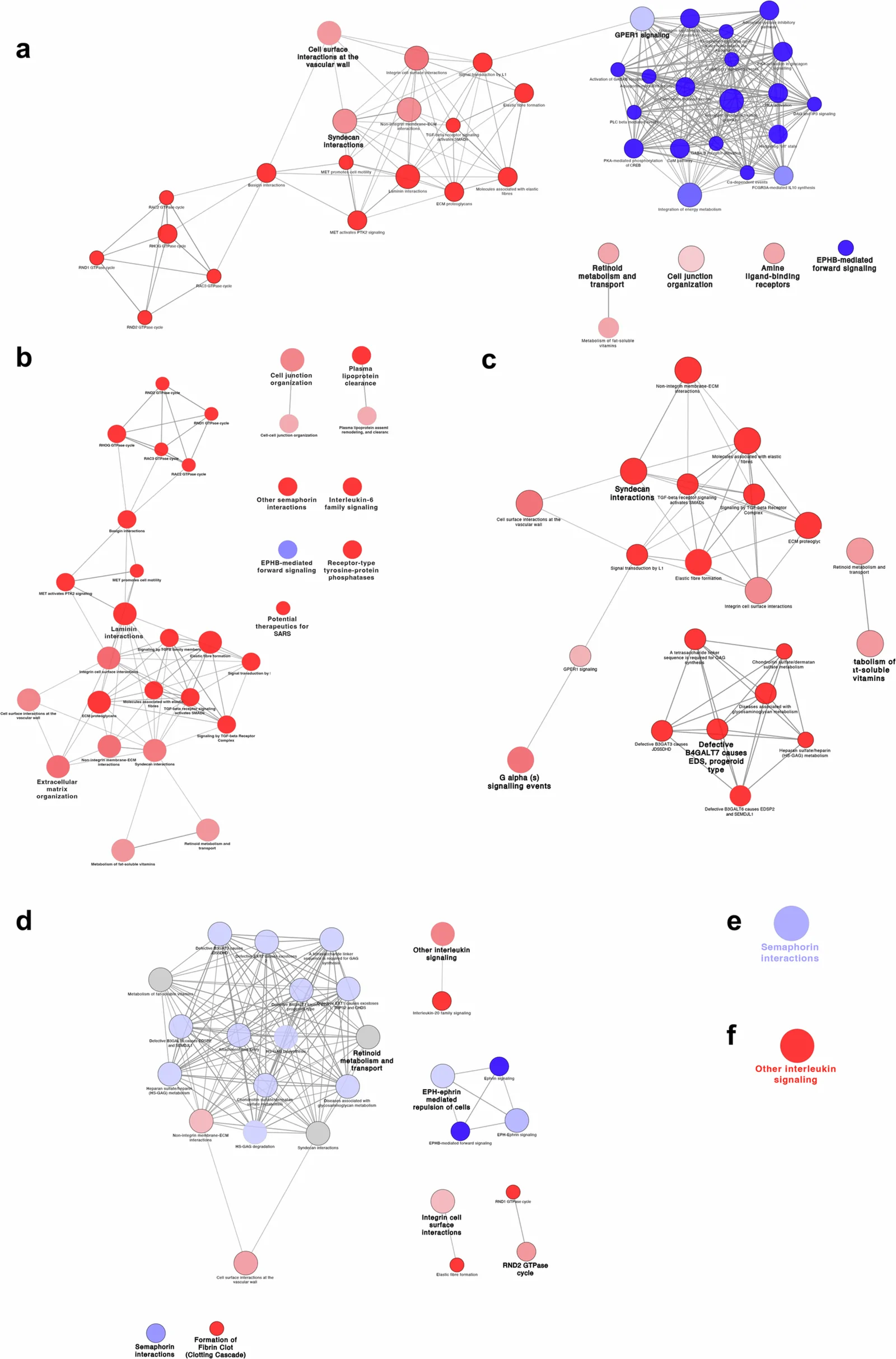
Reactome analysis. **a**, Fundus receptor DEG reactome (organoid vs MRA). **b**, Body receptor DEG reactome (organoid vs MRA). **c**, Antrum receptor DEG reactome (organoid vs MRA). **d**, Fundus receptor DEG reactome (SRA vs MRA). **e**, Body receptor DEG reactome (SRA vs MRA). **f**, Antrum receptor DEG reactome (SRA vs MRA).

Subsequently, we performed ligand–receptor couple analyses to identify signalling pathways arising in MRAs compared with simpler organoid systems that might be responsible for paracrine effects inducing cell differentiation. First, the identified DEG receptors highlighted marked differential expression between organoids and MRAs of the different regions (Fig. 3d). Of particular interest was the reduced MRA expression of the stem cell markers *LGR5* and *TROY* (also known as *TNFRSF19* in body and fundus, where it is physiologically expressed), suggesting a loss of stemness in favour of more differentiated phenotypes, as was previously reported in primary gastric organoids^7^. *NOTCH3*, involved in gastric stem cell maintenance and the control of differentiation pathways of various cell types (such as parietal, chief and foveolar cells) was upregulated in antrum MRAs. Of note, body MRAs presented decreased expression of *FGFR4*, which mediates cell proliferation in gastric cancer^26^. Additionally, the protein tyrosine phosphatase receptor *PTPRF*, which is responsible for regulating cell differentiation, was more expressed in MRAs than organoids. *SSTR1* was upregulated in all MRA regions (Extended Data Fig. 3g). Interestingly, we could observe an upregulation of ligand–receptor pairs in the antrum. It has been reported that the Notch pathway regulates epithelial cell differentiation in the antral stomach through *NOTCH* receptors by promoting progenitor cell proliferation and inhibiting differentiation^27^. Indeed, we identified the upregulation of *NOTCH3* and *DLL4*, which play a crucial role in regulating fate decision, proliferation and differentiation. Moreover, we could observe SLPI (contributing to maintenance of gastric mucosal integrity), AREG (involved in cell proliferation and tissue repair) and BMP2 ligands (contributing to parietal cell differentiation) upregulated in MRAs (Fig. 3d). We then validated the pathway changes between organoid and MRA conditions, by analysing how DLL4–NOTCH1–NOTCH3 (ligand–receptor–receptor) and FGF11–FGFR4 (ligand–receptor) evolve from organoids to assembloids and then to disassembled assembloids. The results showed how NOTCH and fibroblast growth factor (FGF) pathways change when the organoids self-assemble into MRAs, and were restored to their original expression levels after MRA disaggregation into organoids, suggesting an effect of the three-dimensional (3D) multi-regional self-assembly (Extended Data Fig. 3h). Functional enrichment analysis within the Reactome database confirmed that other ligands were involved in extracellular matrix (ECM) remodelling (Extended Data Fig. 4).

To exclude that the pathway changes were ascribed to a different ECM environment (Matrigel in organoids versus collagen I in MRAs), we performed ligand–receptor analysis on SRAs versus MRAs (both in collagen I). *SSTR1* was found to be upregulated in MRAs. In MRAs, *LRG5* (body) and *TROY* (in both the fundus and body) were downregulated. *FGFRL1* (responsible for tuning other FGF signalling pathways) was downregulated in MRAs (Extended Data Fig. 3i). The ligand–receptor pair analysis revealed changes in key pathways, including increased expression of the antrum receptor *ADRB2* (involved in lipid and glycogen metabolism), the ligands *HBEGF* and *AREG* (both involved in cell differentiation and tissue regeneration) and the antrum ligand *RBP4* (involved in retinol transport in glucose metabolism and insulin sensitivity). These changes were more evident in MRAs compared with SRAs (Fig. 3e). Taken together, these data confirm not only that there is an increase in system complexity from organoids to SRAs, but also that the multi-regionality feature of MRAs induces a relevant change in gastric pathway expression.

### The MRA system promotes parietal cell differentiation and function

**Fig. 4:**
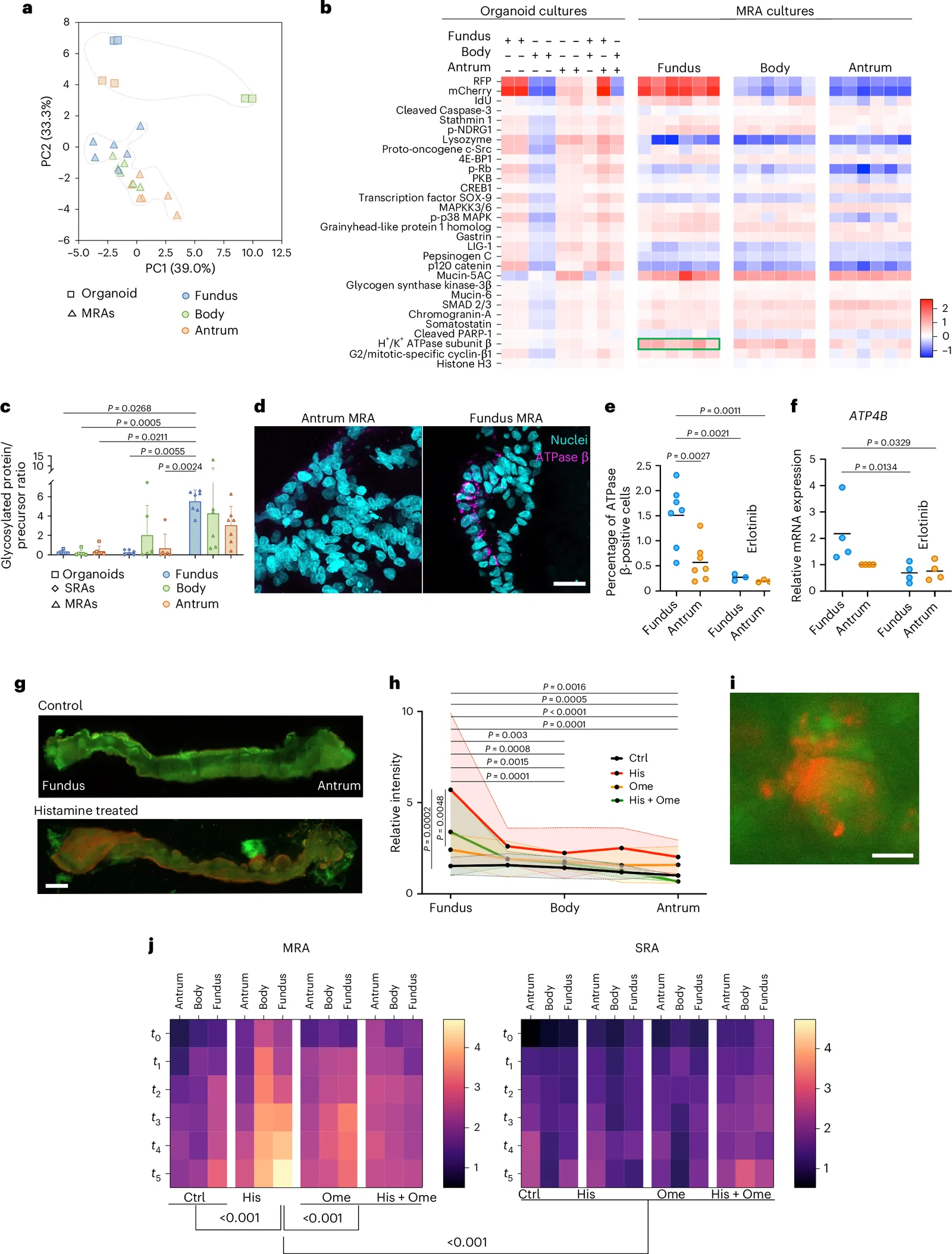
Mass cytometry and functional analysis of the MRA. **a**, PCA of TOBis MC data from fundus (blue), body (green) and antrum (red) organoids (squares) and MRAs (triangles) (*n* = 2 for organoids; *n* = 6 for MRAs; patient 1). **b**, Heatmap showing the abundances of selected markers by TOBis MC analysis in organoid cultures compared with MRA cultures. The green box highlights ATPase β expression in fundus MRAs. **c**, ATPase β protein expression in the fundus, body and antrum in organoids, SRAs and MRAs using WES analysis, displayed as the quantification of glycosylated protein over precursor. Single data points are displayed. The error bars represent means ± s.d. (*n* = 6 for organoids (patients 2, 3 and 4); *n* = 6 for SRAs (patients 2, 3 and 4); *n* = 7 for MRAs (patients 2, 3 and 4)). Statistical significance was determined by Kruskal–Wallis test for comparison against the MRA fundus group. **d**, Antrum and fundus MRA sections stained for ATPase β (magenta) and nuclei (cyan; Hoechst). Scale bar, 50 μm. **e**, Percentage of ATPase β-positive cells in the antrum and fundus in control MRAs versus MRAs treated with 20 nM erlotinib. Statistical significance was determined by one-way analysis of variance (ANOVA) (*n* = 7 for the control; *n* = 3 for the treatment; patients 3 and 4). **f**, Relative *ATP4B* expression, normalized to that of *GAPDH*, in the antrum and fundus in control MRAs versus MRAs treated with 20 nM erlotinib (*n* = 4; two patients). Statistical significance was determined by Kruskal–Wallis test for comparison with the fundus group. **g**, Acridine orange staining of MRAs with or without 1 h of histamine treatment (100 μM). The emission wavelengths were 500–550 and 600–650 nm. Scale bar, 500 μm. **h**, Acridine orange 650 nm/550 nm ratio quantification of the fundus, body and antrum of MRAs, normalized to an antrum control. Black, red, yellow and green represent the control (Ctrl), histamine treatment (100 μM; His), omeprazole treatment (100 μM; Ome) and histamine + omeprazole treatment (100 μM). The circles indicate the average of single-patient data points ± s.d. (*n* > 3 technical replicates; patients 3 and 4). Statistical significance was determined by one-way ANOVA. **i**, Acridine orange image of green and red channels of a single cell in the fundus upon histamine stimulation. Scale bar, 20 μm. **j**, Heatmap of acridine orange 650 nm/550 nm ratio timelapse quantification of single cells from the fundus, body and antrum in SRAs and MRAs at six timepoints (every 10 min for 1 h). Same experimental conditions as in **h**. The data points indicate an average of 12–31 cells per point (patients 3 and 4). Statistical significance was determined by one-way ANOVA at the *t*_5_ timepoint (adjusted *P* value MRA-HisF versus MRA-Ctrl all <0.01, MRA-HisF versus MRA-Ome A,B and MRA-HisOme all <0.001, MRA-HisF versus SRA-Ctrl, His, Ome all and HisOme A <0.001, MRA-HisF versus SRA-HisOme B,F <0.05. For single *P* values, refer to the associated source data. Source Data

**Extended Data Fig. 5:**
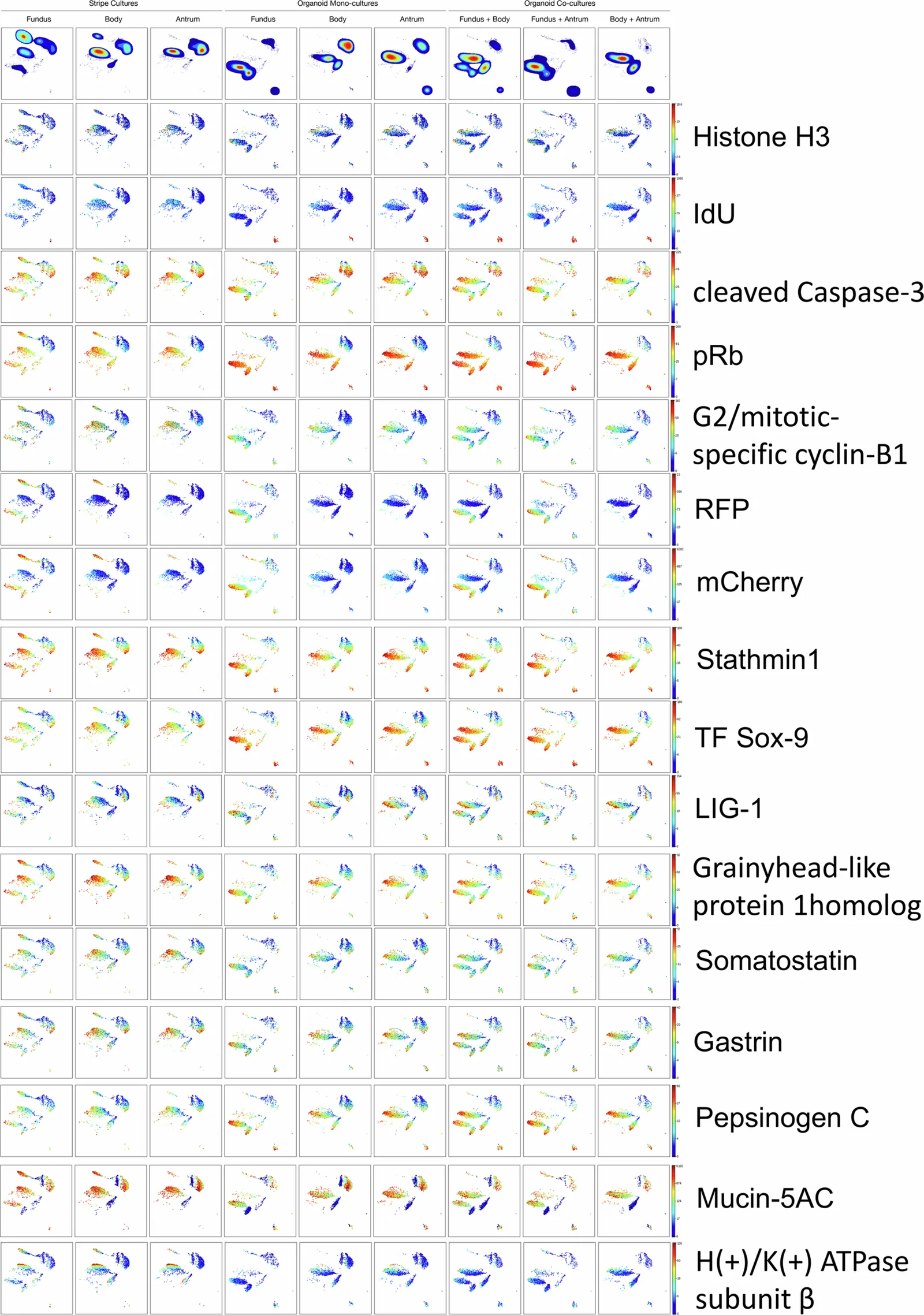
UMAPs of the analysed markers. UMAPs of the analysed markers based on TOBis MC analysis in organoid cultures, organoid co-cultures and MRA.

**Extended Data Fig. 6:**
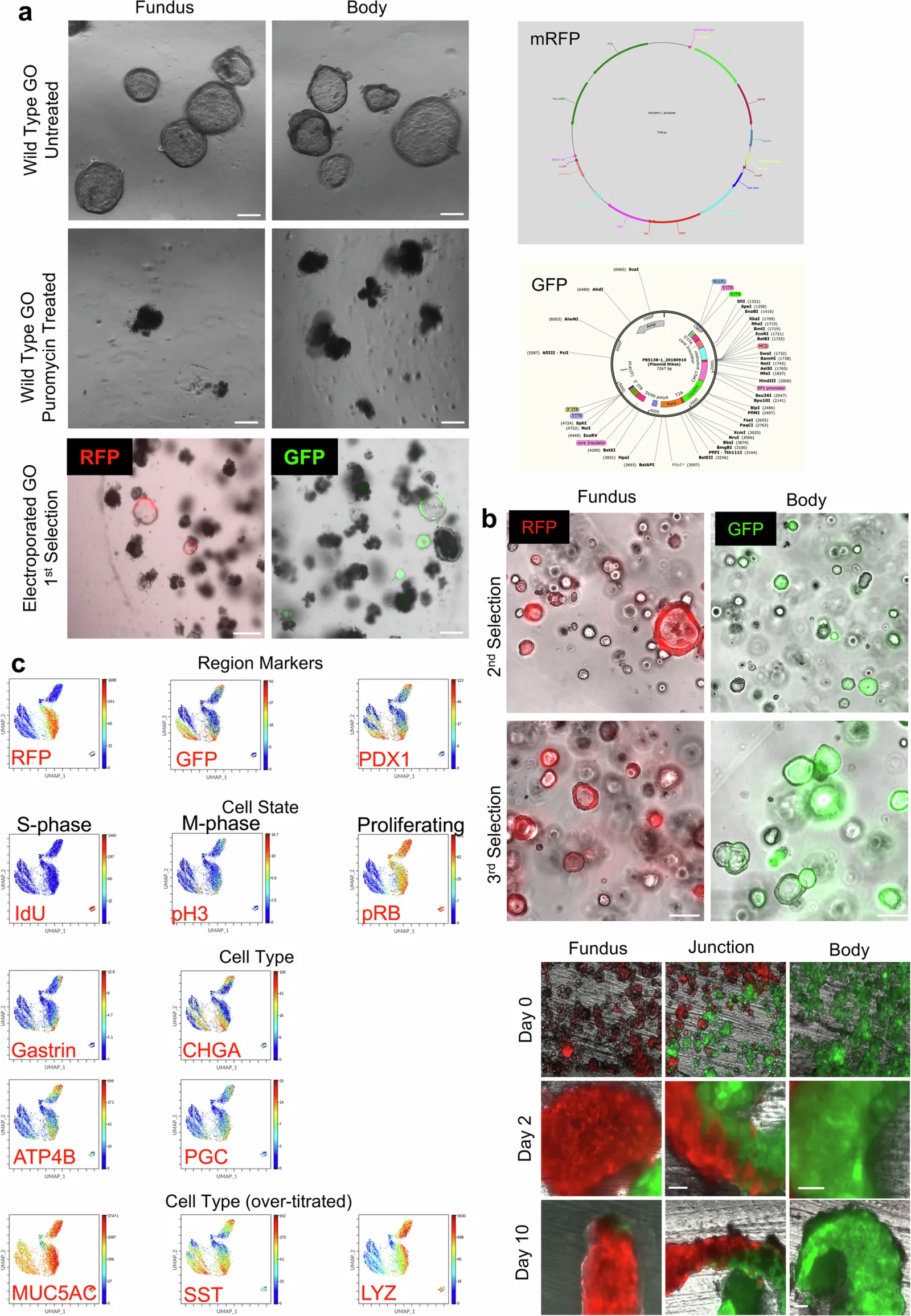
TOBis MC cell line preparation and preliminary analysis. **a**, Plasmid maps and generation of reporter RFP-fundus and GFP-body organoid lines by electroporation. Scale bars 200 μm. **b**, 2^nd^ and 3^rd^ selections of electroporated GOs at day 7 of passage (top). Fusion of adjacent regions in MRA (bottom). RFP-expressing fundus organoids, GFP-expressing body organoids. Scale bars 200 μm. **c**, Optimisation of antibody staining of MRA tubes for CyTOF.

Having established an enhanced morphological complexity with increased gastric epithelial differentiation and cross-talk, we then focused on assessing the gastric functionality of MRAs. We performed thiol organoid barcoding in situ mass cytometry (TOBis MC)^28,29^ to characterize the post-translational/signalling profile of MRAs and organoids at single-cell resolution—an optimized technique for high-dimension panels^30,31^. We compared a panel of 30 proteins in the three MRA regions and in individual organoid cultures (Extended Data Fig. 5). To aid cell-type-specific analysis, fundic organoids were labelled with red fluorescent protein (RFP) and body organoids were marked with green fluorescent protein (GFP) (Extended Data Fig. 6a,b). Antral organoids were not labelled due to the presence of PDX1—a marker that allows their unique identification (Extended Data Fig. 6c). PCA of protein signals clearly resolved organoid and MRA signalling (Fig. 4a).

**Extended Data Fig. 7:**
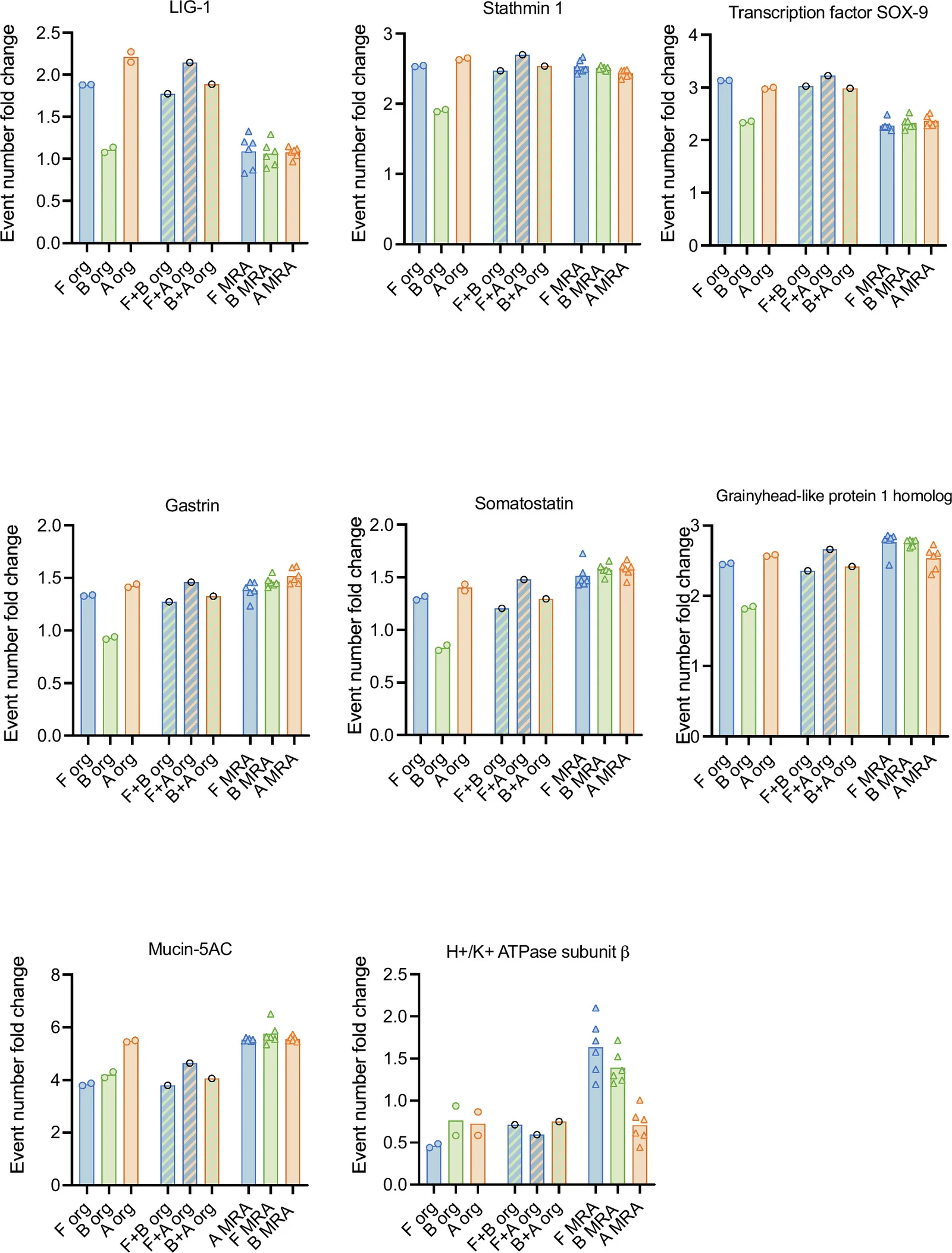
TOBis MC normalized reads for selected proteins. LRIG1, STATHMIN, SOX9, GAST, SST, GHRELIN, MUC5AC, ATPase β normalized reads plotted for following conditions: organoid cultures (Fundus, Body, Antrum), organoid co-cultures (F + B = Fundus and Body; F + A = Fundus and Antrum; B + A = Body and Antrum), MRA (Fundus, Body, Antrum). Mean and single data points displayed (n = 2 for organoids, n = 1 for organoid co-cultures, n = 6 for MRA). Source Data

Overall, the expression of stem cell markers, including SOX9, LRIG1 and Stathmin1, was decreased in MRAs, whereas the expression of some differentiation markers was increased (Fig. 4b)^32^. Specifically, antral-specific markers, such as SST and GAST, were mostly found in the antral end of MRAs. Interestingly, parietal cell protein ATPase β expression was significantly higher in MRAs compared with organoids, with physiological expression on the fundus and body of assembloids. Of note, the mere co-culture of organoids in adherent Matrigel droplets was not enough to induce a shift of expression from an organoid signature to an MRA one. Indeed, plotting the normalized reads corresponding to the different conditions, we demonstrated a substantialdifference between MRAs and organoids and between MRAs and organoid co-culture (Extended Data Fig. 7). The stem cell markers LRIG1, stathmin and SOX9 were all downregulated in MRAs compared with the other conditions, underlying the promotion of cell differentiation in the assembloids. In contrast, the gastric differentiation markers GAST, SST, ghrelin and MUC5AC were all upregulated in MRAs compared with the other conditions. Also, GAST was regionally overexpressed in the MRA antrum compared with the fundus; ghrelin was more expressed in the MRA fundus and body compared with the antrum; and mucin was equally expressed through the MRA regions, demonstrating the model’s high fidelity to native tissue with regionality preserved. Moreover, ATPase β protein was significantly overexpressed in MRAs compared with both single organoids and organoid regional duplets (organoid co-cultures), and its expression was confirmed to be higher in the MRA fundus compared with the antrum (Extended Data Fig. 7).

To investigate whether MRAs are able to produce and secrete gastric acid, the glycosylated functional form of ATPase β (as found in parietal cell membranes) was evaluated by Simple Western (WES) protein analysis. The ATPase β initial precursor (47–53 kDa) undergoes glycosylation into functional proteins from 60–80 kDa that are transported to the membrane for acid production^33,34^. Strikingly, we verified a higher ratio of glycosylated ATPase β/precursor in the MRA fundus and body compared with its antral end and and each regional organoid and SRA (Fig. 4c).

**Extended Data Fig. 8:**
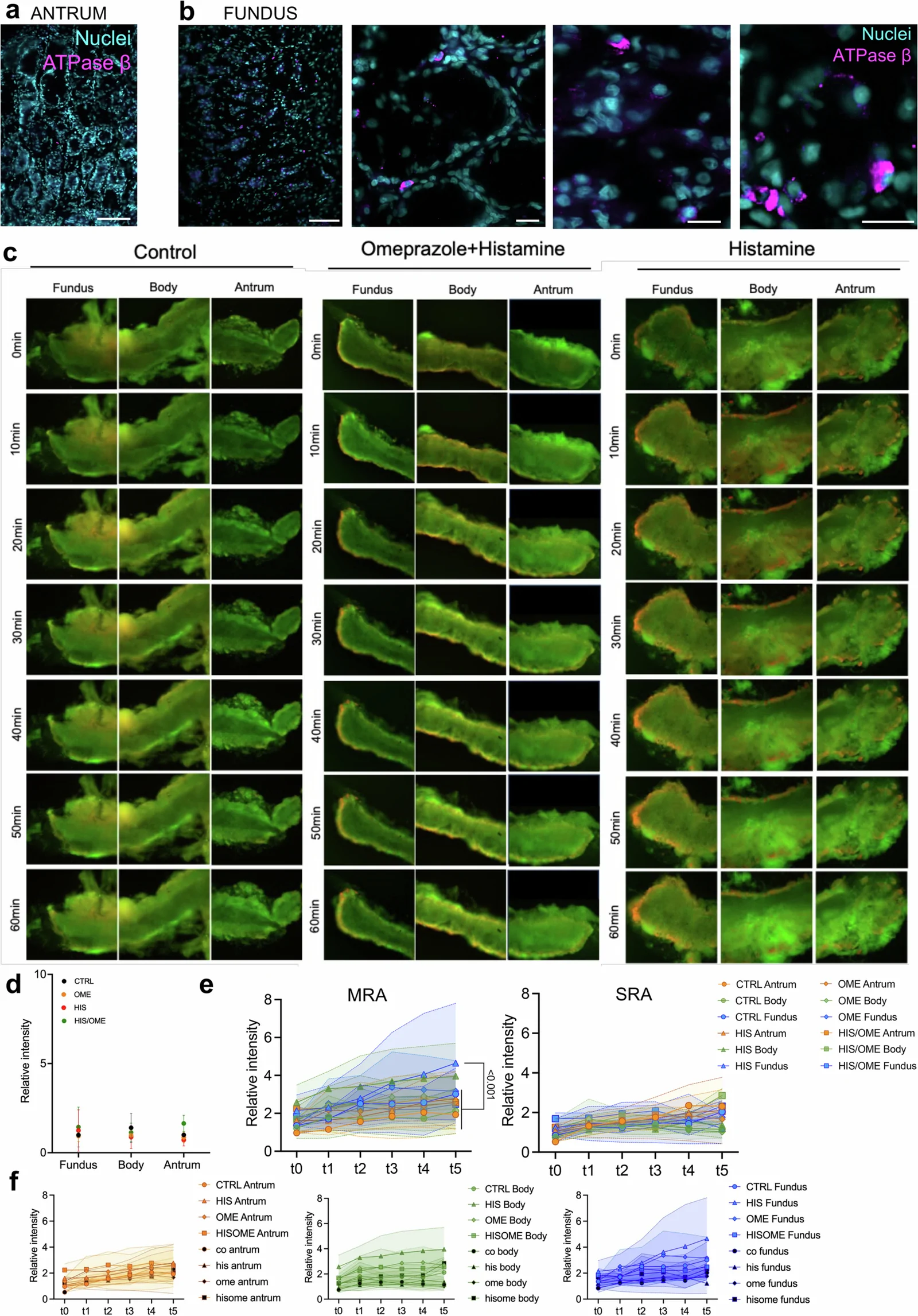
MRA functional analysis. **a**, Immunofluorescence of sectioned antrum tissue showing ATPase β in magenta, and Nuclei (Hoeachst) in cyan. Scale bar 200μm. **b**, Immunofluorescence of sectioned fundus tissue showing ATPase β in magenta, and Nuclei (Hoeachst) in cyan. Scale bar 200μm in the first panel, 50μm in the following. **c**, Timelapse frames of Acridine orange staining (10 μM) of Fundus, Body, and Antrum in MRA, Control, Histamine treatment (100 μM), Histamine+Omeprazole (100 μM). Images acquired every 10 mins. **d**, Acridine orange 650/550 nm ratio quantification of Fundus, Body, and Antrum in SRA, normalized to MRA Antrum-control. Control (black), Histamine treatment (100 μM, red), Omeprazole treatment (100 μM, orange), Histamine+Omeprazole (100 μM, green). Circles indicate average of single patient data points, mean ± SD (n = 4, 2 patients). One-way ANOVA, NS. **e**, Acridine orange 650/550 nm ratio timelapse quantification of single cells from Fundus, Body, and Antrum in SRA and MRA at 6 timepoints (every 10 min for 1 h). Control (circles), Histamine treatment (100 μM – triangles), Omeprazole (100 μM – rombi), Histamine+Omeprazole (100 μM – squares). Data points indicate average of 12 to 31 cells per each point. mean ± SD (2 patient). One-way ANOVA of *t*_5_ timepoint (for single p-value, refer to the source data for Fig. 4j). **f**, Acridine orange 650/550 nm ratio timelapse quantification of single cells in SRA (black filled symbols) and MRA (coloured symbols) at 6 timepoints (every 10 min for 1 h) subdivided in three graphs per region: Antrum (orange), Body (green), and Fundus (blue). Control (circles), Histamine treatment (100 μM – triangles), Omeprazole (100 μM – rombi), Histamine+Omeprazole (100 μM – squares). Data points indicate average of 12 to 31 cells per each point. mean ± SD (2 patient). Source Data

We further confirmed the presence of ATPase β-positive cells by immunostaining (Fig. 4d and Extended Data Fig. 8a,b), with the fundus cell percentage significantly higher than for the antral end (Fig. 4e). To verify that the parietal cells we observed were regulated by the epidermal growth factor (EGF) and bone morphogenetic protein (BMP) pathways^16^, we imposed an EGF^−^ BMP^−^ culture condition, to observe a decrease in the number of ATPase β-positive cells^16^. The EGF receptor inhibitor erlotinib (20 nM) was used to treat the MRAs during the assembly period (days 4–10). This resulted in a significant decrease in the number of positive cells and a corresponding decrease in *ATP4B* mRNA expression (immunofluorescence in Fig. 4e and RT-qPCR in Fig. 4f).

At the functional level, the acid production mediated by the glycosylated form of ATPase β in the complex of the ATPase H^+^/K^+^ pump was evaluated by live imaging with acridine orange, which shows a λ emission shift when the pH is ≤3.5. We compared four conditions: (1) a control condition; (2) 1 h of treatment with histamine (an acid secretion stimulator); (3) 2 h of treatment with omeprazole (a chemical inhibitor of the pump); and (4) treatment with both histamine and omeprazole. Under control conditions, as well as for the omeprazole treatment group, we observed no significant differences in the three regions of the MRA. In contrast, the histamine treatment induced an increase in the fluorescence intensity ratio, indicating higher acid secretion. Notably, the combination of histamine and omeprazole showed a signal reduction compared with the histamine treatment alone (Fig. 4g,h). Timelapse images of the experiment can be found in Extended Data Fig. 8c and Supplementary Videos 4–6. Strikingly, when performing this analysis on fundus, body and antrum SRAs, a low relative intensity was measured and no significant difference was identified (Extended Data Fig. 8d), showing a lack of functionality compared with MRAs. Moreover, we examined single-cell progression through 1-h timelapse microscopy in SRAs and MRAs, comparing the four previously analysed experimental conditions (control, histamine, omeprazole and histamine + omeprazole). Acridine orange accumulation was observed in a canalicular-type pattern in parietal cells, in a similar fashion to what was previously observed^10^ (Fig. 4i). In MRAs, we detected a signal ratio increase across the six timepoints, with a higher signal in cells on the fundic side, showing the maximum ratio upon histamine stimulation. Meanwhile, SRA single-cell analysis resulted in a significantly lower ratio compared with the fundus MRA treated with histamine (Fig. 4j and Extended Data Fig. 8e,f). Taken together, these data suggest that the MRA system favours enhanced gastric parietal differentiation with the appearance of complex gastric-specific functionality (histamine-responsive acid secretion).

**Extended Data Fig. 9:**
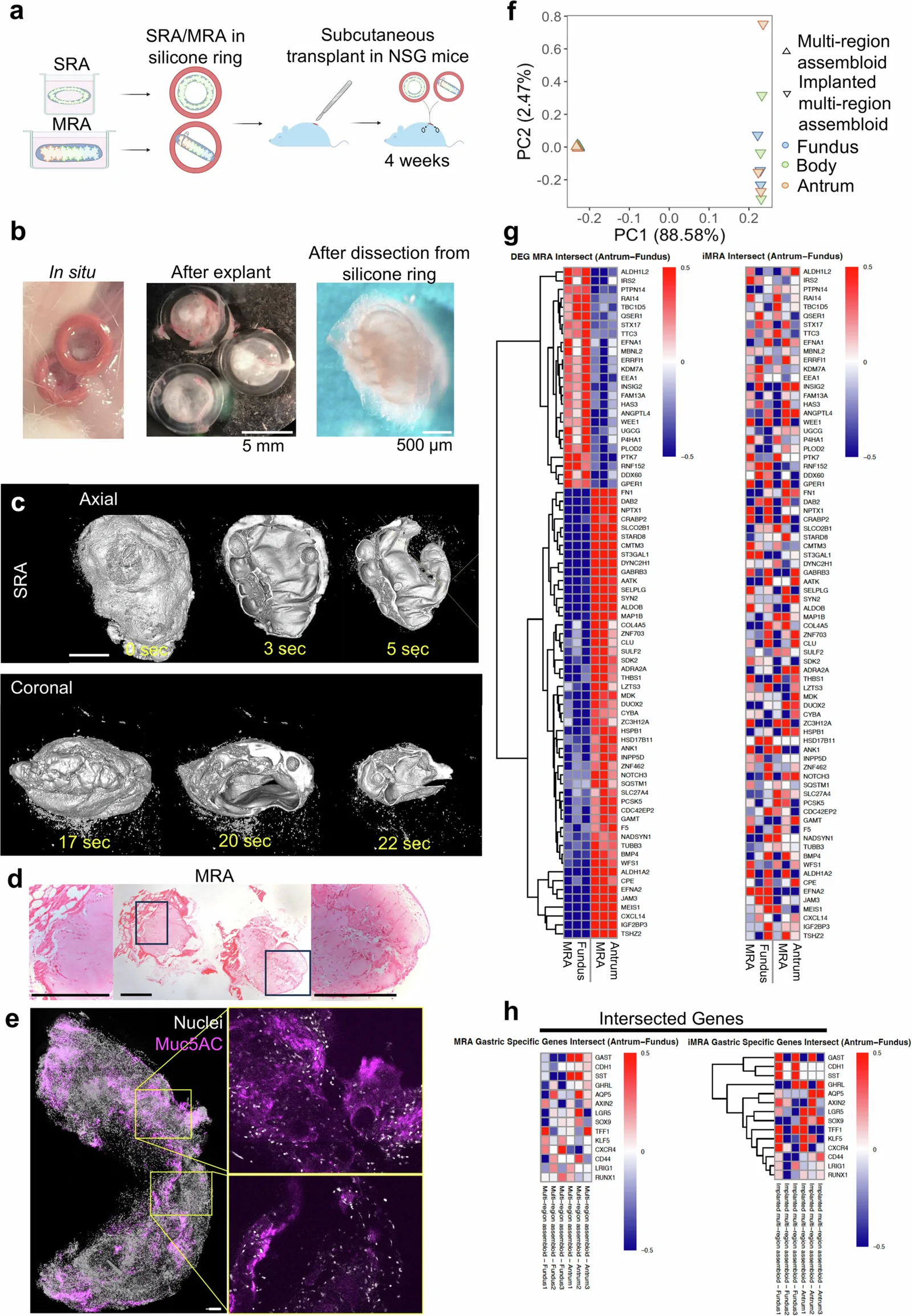
In vivo implantation of single- and multi-regional gastric assembloids. **a**, Schematic representation of assembloid implantation workflow. **b**, Postimplantation assembloid harvesting: sub-cutaneous location (left), after explanation (central, scale bar 5 mm), isolated assembloid (right, scale bar 500 μm) (n = 3, patient 1). **c**, Microfocus computed tomography (Micro-CT) of transplanted SRA from axial and coronal view. Scale bar 500 μm. **d**, H&E staining of iMRA slice. Scale bar 500 μm. **e**, Whole mount 3D reconstruction of gastric iMRA showing nuclei in white (Reddot), Mucin 5AC (MUC5AC) in magenta. Scale bar 100 μm. **f**, Principal component analysis (PCA) of RNA-sequencing samples from Fundus (blue), Body (green), and Antrum (red) in MRA (triangle) and postimplantation MRA (iMRA, inverted triangle). (n = 3, patient 1). **g**, Hierarchical clustering of DEGs (FDR < 0.05, fold change > 1.5) between Antrum and Fundus in MRA (left), and expression of the corresponding genes in iMRA (right). (n = 3, patient 1). Color bar refers to centered log2(CPM + 1). **h**, Heatmaps of selected gastric specific genes differential expression between Antrum and Fundus in MRA or in iMRA. (n = 3, patient 1). Panel **a** created with BioRender.com. Source Data

Subsequently, the behaviour of the assembloid was evaluated in vivo upon subcutaneous implantation into immunodeficient NSG mice (Extended Data Fig. 9a). Four weeks after transplantation, the explants were viable and surrounded by host mesenchyme and vasculature (Extended Data Fig. 9b). The explants were characterized by microfocus computed tomography (micro-CT) and whole-mount immunofluorescence. Three-dimensional reconstructions of the SRA micro-CT demonstrated preservation of a shared lumen and basal budding structures (Extended Data Fig. 9c and Supplementary Videos 7 and 8). Haematoxylin and eosin and immunofluorescence analyses on MRA explants confirmed the presence of large, shared lumens, as well as luminal secretion of MUC5AC (Extended Data Fig. 9d,e), suggesting maintenance of gastric epithelial cell identity. RNA-seq was performed on post-implantation MRAs (iMRA). PCA between MRAs and iMRAs showed a distinctive separation between the two groups of samples (Extended Data Fig. 9f). Further analysis comparing antral and fundic expression within the assembloids showed notable heterogeneity in iMRAs, ascribable to the biological variability in an in vivo environment (Extended Data Fig. 9g). Notably, the DEGs analysis comparing antrum versus fundus in MRAs and iMRAs showed decreased stem cell marker expression in the iMRA fundus compared with its antrum, suggesting higher fundic phenotype differentiation in vivo. Additionally, further extrapolated DEGs among a curated list of gastric-specific genes of the antrum versus fundus in MRAs and iMRAs confirmed the previous observation in the fundus, and functional markers, such as aquaporin 5, were physiologically upregulated in the antrum side of iMRAs (Extended Data Fig. 9h).

### MRA model of PMM2–HIPKD–IBD gastric manifestation

Endoscopic biopsies from two patients with PMM2–HIPKD–IBD were used to derive promoter variant *PMM2* (hereafter referred to as *PMM2*) gastric organoids and generate a *PMM2*-MRA model.

**Fig. 5:**
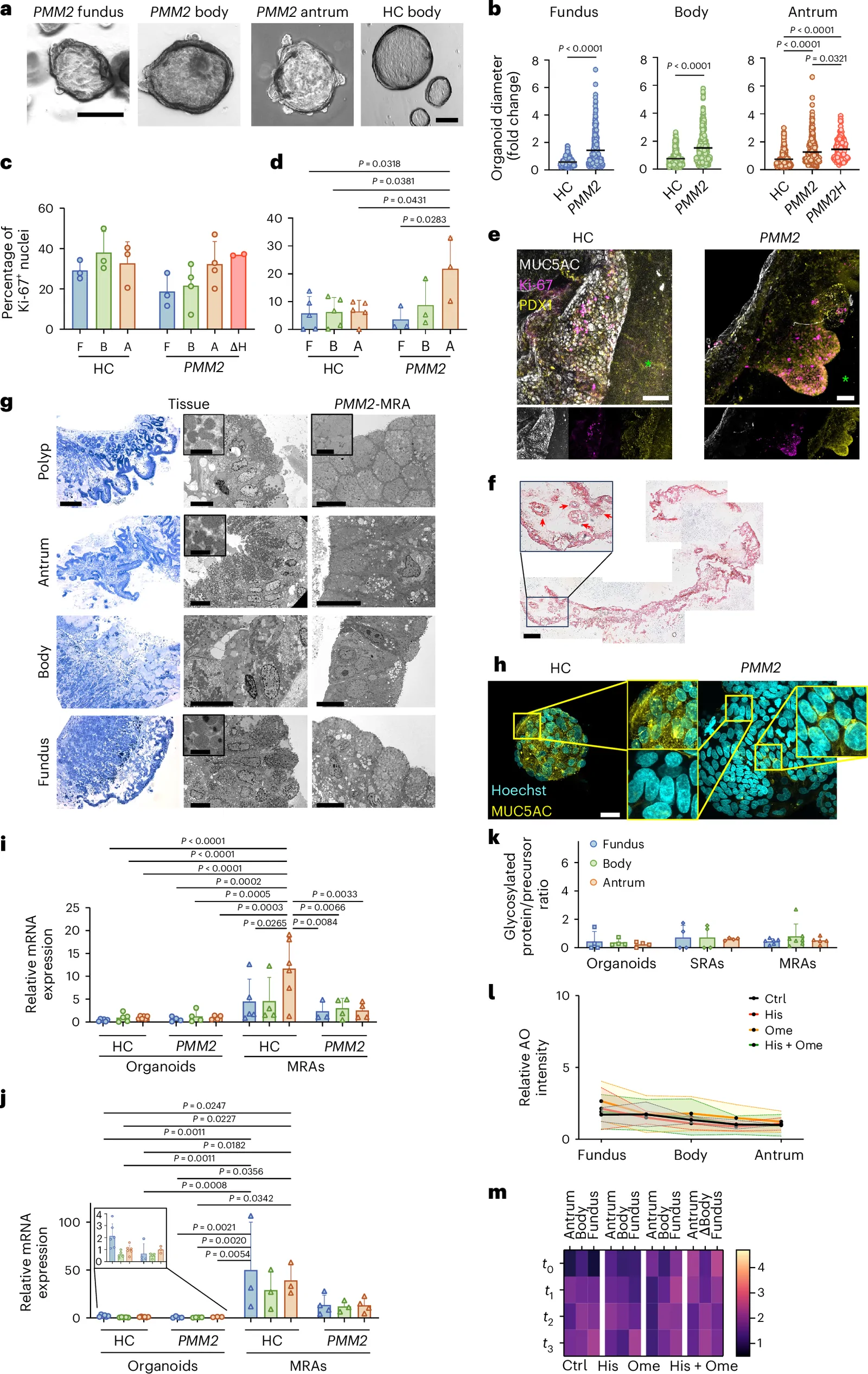
Disease modelling of the *HNF4A*/*PMM2* genetic condition with gastric MRAs. **a**, Brightfield images of *PMM2* fundus, body and antrum organoids compared with healthy control (HC) body organoids. Scale bars, 100 μm. **b**, Full-grown organoid diameters were measured in three HCs and a minimum of two *PMM2* organoid lines. Relative fold changes are shown compared with the average for *PMM2* antrum. The data points represent single organoids and the means are shown as a bar. *PMM2*H represents organoids from hyperplastic polyp. Statistical significance was determined by *t*-test analysis for the fundus and body and one-way ANOVA for the antrum. **c**, Ki-67^+^ percentages in whole-mount-stained HC organoids (*n* = 3) versus *PMM2* organoids (*n* = 4). The data points represent the single-patient average and the error bars represent means ± s.d. Statistical significance was determined by one-way ANOVA. No significant differences were found. **d**, Ki-67^+^ percentages in whole-mount-stained MRA regions (*n* = 5 for HCs (three patients) and *n* = 3 for *PMM2* (two patients)). The data represent means ± s.d. Statistical significance was determined by one-way ANOVA. F, fundus; B, body; and A, antrum. **e**, Whole-mount immunostaining of the antral region of a HC MRA and a *PMM2*-MRA for Ki-67 (magenta), PDX1 (yellow) and MUC5AC (white). The asterisk identifies the luminal side of the MRA. Scale bars, 50 μm. **f**, Imaging tiling of a haematoxylin-and-eosin-stained section of a *PMM2*-MRA. The red arrows indicate cell aggregates. Scale bar, 500 μm. **g**, Toluidine blue staining (scale bar, 200 μm) and matching TEM images of *PMM2* tissue samples and TEM images of *PMM2*-MRAs (scale bars, 10 μm), with magnifications of mucin granules (scale bars, 2 μm; *n* = 1). **h**, Whole-mount staining of HC and *PMM2* organoids for nuclei (cyan) and MUC5AC (yellow). Scale bar, 20 μm. **i,** *MUC5AC* RT-qPCR data (*n* = 6 for HC organoids (three patients), *n* = 4 for *PMM2* organoids (two patients) *n* = 5 for HC MRAs (three patients) and *n* = 4 for *PMM2* MRAs (two patients)). **j**, *ATP4B* RT-qPCR data (*n* = 6 for HC organoids (three patients), *n* = 4 for *PMM2* organoids (two patients), *n* = 3 for HC MRAs (three patients) and *n* = 4 for *PMM2*-MRAs (two patients)). In **i** and **j**, the data represent means ± s.d. and statistical significance was determined by one-way ANOVA. Blue, green and red data points represent samples from the fundus, body and antrum, respectively. **k**, WES analysis of the ATPase β protein ratio (glycosylated/precursor) in fundus, body and antrum *PMM2* organoids, MRAs and SRAs. Single data points are shown, where circles represent organoids (*n* = 4; two patients), rhomboids represent SRAs (*n* = 6; two patients) and triangles represent MRAs (*n* = 6; two patients). Statistical significance was determined by Kruskal–Wallis test. No significant differences were found. **l**, Acridine orange (AO) 650 nm/550 nm *PMM2*-MRA ratio quantification (fundus, body and antrum), relative to the antrum control. The treatments included histamine (100 μM), omeprazole (100 μM) and histamine + omeprazole (100 μM). The circles indicate the average of single patients and the data represent means ± s.d. (*n* = 3; two patients). **m**, Acridine orange 650 nm/550 nm ratio timelapse quantification of single cells (fundus, body and antrum) in *PMM2*-MRAs at four timepoints (every 20 min for 1 h). The treatment groups were as in **l**. The data points indicate an average of 12 cells (two patients). Statistical significance was determined by one-way ANOVA for the *t*_3_ timepoint. Source Data

**Extended Data Fig. 10:**
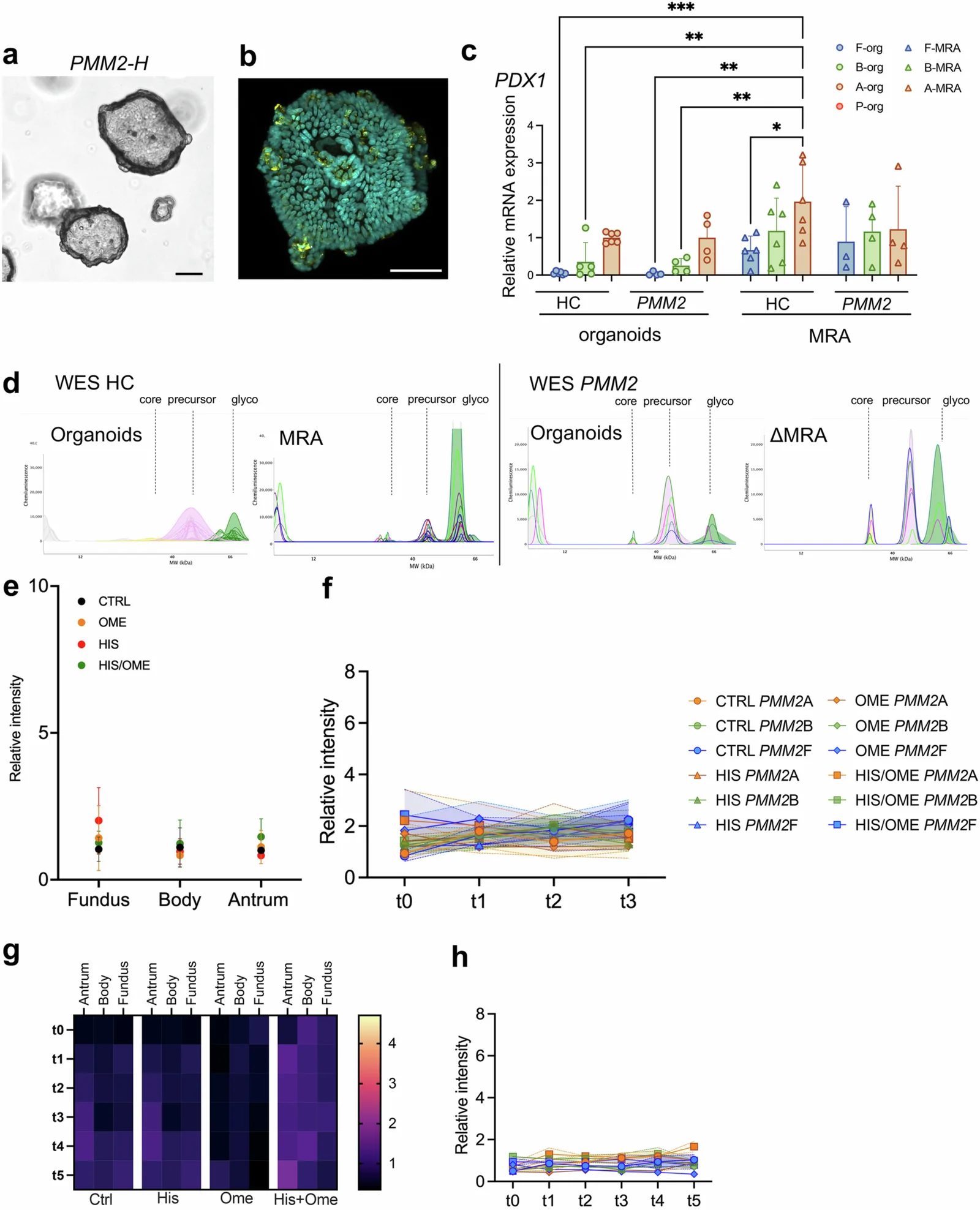
Disease modelling. **a**, Bright field image of organoids derived from hyperplastic region of patient affected by mutation (*PMM2*), showing the formation of spherical organoids with visible buddings within 7 days starting from single cells. Scale bar 100 μm. **b**, Whole mount immunostaining of *PMM2*-H organoids (Scale bar 100 μm). **c**, RT-qPCR for *PDX1*, Organoids vs MRA, HC vs *PMM2* (HC organoids n = 5, 3 patients; *PMM2* organoids n = 4, 2 patients; HC MRA n = 6, 3 patients; *PMM2*-MRA n = 4, 2 patients). One-way ANOVA. **d**, Collective graphs of the peaks identified by the WES analysis (Compass Software v.6.1.0) in Organoids vs MRA of HC on the left (n = 2 per 3 patients organoids, n = 3 per 3 patients MRA) and *PMM2* on the right (n = 2 technical, 1 patient only): core protein = yellow area; precursor = pink area; glycosylated protein = green area. **e**, Acridine orange 650/550 nm ratio quantification of Fundus, Body, and Antrum in *PMM2*-SRA, normalized to *PMM2*MRA Antrum-control. Control (black), Histamine-treated (100 μM, red), Omeprazole-treated (100 μM, orange), Histamine+Omeprazole (100 μM, green). Circles indicate average of single patient data points, mean±SD (n = 2, 2 patients). One-way ANOVA. **f**, Acridine orange 650/550 nm ratio timelapse quantification of single cells from Fundus, Body, and Antrum in *PMM2*-MRA at 4 timepoints (every 20 min for 1 h). Control (circles), Histamine-treated (100 μM-triangles), Omeprazole (100 μM–rombi), Histamine+Omeprazole (100 μM–squares). Data points indicate average of 12 per each point (2 patients). One-way ANOVA on *t*_3_ timepoint. **g**, Heatmap of acridine orange 650/550 nm ratio timelapse quantification of single cells from Fundus, Body, and Antrum in *PMM2*-SRA at 6 timepoints (every 10 min for 1 h). Control, Histamine treatment (100 μM), Omeprazole (100 μM), Histamine+Omeprazole (100 μM). Data points indicate average of 6 cells per each point. mean±SD (1 patient). One-way ANOVA of *t*_5_ timepoint. **h**, Acridine orange 650/550 nm ratio timelapse quantification of single cells from Fundus, Body, and Antrum in *PMM2*-SRA at 6 timepoints (every 10 min for 1 h). Control (circles), Histamine treatment (100 μM-triagles), Omeprazole (100 μM-rombi), Histamine+Omeprazole (100 μM-squares). Data points indicate average of 6 cells per each point (1 patient). One-way ANOVA of *t*_5_ timepoint. Source Data

Fundus, body and antrum organoids were successfully derived from endoscopic biopsies of both patients (Fig. 5a). For one of the two patients, a biopsy was collected directly from a hyperplastic polyp itself, allowing the derivation of organoids from this fourth anatomic location (Extended Data Fig. 10a,b). Upon initial morphological comparison with organoids derived from a healthy control, both organoid conditions shared a cystic and round appearance. However, *PMM2* organoids exhibited a higher abundance of bud-like protrusions at similar passage (Fig. 5a) and were significantly larger compared with the healthy control counterpart (Fig. 5b). Although the numbers of Ki-67^+^ proliferative cells were comparable among all organoid lines (Fig. 5c), for *PMM2*-MRA we identified focal regions with significantly enhanced proliferation, particularly within the antral side (Fig. 5d). This phenotype was even more pronounced when analysing polyp-derived organoids, where whole PDX1^+^ protruding buds were highly positive for Ki-67 (Fig. 5e). Haematoxylin and eosin staining of *PMM2*-MRA slices revealed the presence of cell aggregates exclusively on one side of the MRA (Fig. 5f). These structures, representing hyperplastic protrusions, mirrored the foveolar hyperplasia seen in patient biopsy samples, as highlighted by histopathological examination with toluidine blue (Fig. 5g). TEM analysis of the corresponding tissue biopsies revealed a thinner layer of mucin granules in mucous-producing cells compared with normal conditions. Moreover, mucin granules in the hyperplastic regions were less electron dense, synonymous with a less mature state (Fig. 5g). We observed a lower fluorescence of Mucin 5AC immunostaining in *PMM2* organoids (Fig. 5h) and in the hyperplastic regions of *PMM2*-MRA (Fig. 5e). RT-qPCR indicated a significant increase in the gene expression of *MUC5AC* in MRAs compared with organoids in the healthy control, aligning with an increase in cell differentiation, but this was absent in *PMM2*-MRAs (Fig. 5i).

Subsequently, we focused on ATPase β, given that its functional form relies on its N-glycosylation. The expression of *ATP4B* was increased in MRAs compared with organoids. This increase was less in *PMM2*-MRAs (Fig. 5j). We were able to identify the three protein forms in both the healthy control and the *PMM2* patients in three separate peaks at the indicated molecular weights, noticing an increased amount in precursor for *PMM2*-MRA (Extended Data Fig. 10d). We calculated the ratio of quantified values of glycosylated to precursor protein as an index of N-glycosylation activity. As previously mentioned, in the healthy controls we detected enhanced glycosylation activity in MRAs compared with organoids and SRAs. This was significantly decreased in *PMM2*-MRAs, with an equal ratio among the three subtypes, strongly indicating decreased N-glycosylation in the context of the PMM2 variant (Fig. 5k**)**.

Lastly, histamine stimulation of *PMM2*-MRA did not result in a pH change in acridine orange assay, confirming the reduction in functional glycosylated ATPase β (Fig. 5l and Extended Data Fig. 10e). Acridine orange single-cell quantification was performed on *PMM2* samples as well, showing no sensitivity over time to histamine stimulation (Fig. 5m and Extended Data Fig. 10f,g,h). Overall, these data suggest that the MRA system can model the gastric epithelial dysfunctions observed in patients with *PMM2* mutations.

## Discussion

This study focuses on the establishment of paediatric region-specific gastric organoids and the engineering of these organoids into an MRA system to model the human gastric epithelium.

The stomach is a complex organ with distinct anatomical regions defined as the antrum, body and fundus^25,35^. PDX1 has been identified as a marker for the development of gastric antrum in mice^20^ and is expressed in induced-pluripotent-stem-cell-derived gastric organoids^21^. Recognizing the important patterning role of PDX1 during gastric regionalization^36^, we verified its presence at foetal-stage post-conception week 10, as well as its persistence in gastric tissue derived from infants and children. We showed conservation of PDX1 expression in the antral lines we characterized, confirming the ability of primary derived organoids to maintain the spatial information of the gastric region of origin.

Our results demonstrate that region specificity is maintained in organoid lines. These findings were confirmed by bulk RNA-seq and protein validation of the organoids and were independent from any extrinsic signalling, since organoids from different regions were cultured in the same expansion medium. However, this simpler model still lacks mature gastric functions, such as acid secretion.

Assembloids provide a novel strategy to recapitulate complex tissue interactions in vitro. Multiple organoids can be combined to form more complex models, enabling the study of multiple cell types simultaneously^37^. When we increased the complexity of our system, by building SRAs, we observed a degree of cell maturation, demonstrating a clear advantage over the organoids. However, this SRA model lacks cross-communication between the different stomach regions. The degree of differentiation induced in SRAs was further enhanced in MRAs, due to the biochemical cross-talk between the regions. SRAs and MRAs share the same 3D complexity and ECM composition; therefore, the differences observed in MRAs can be ascribed exclusively to their multi-regionality. We were able to assess the regional marker preservation in each region of our MRA, as well as gastric identity retained with the production of mucins and gastric-specific proteins (that is, somatostatin and gastrin). Our system allows interaction of the three regions with the establishment of a shared lumen, defined apicobasal cell polarization and gastric gland-like protrusions.

Notably, PDX1 expression remained confined to the antrum region, indicating the absence of organoid migration along the assembloid, during organoid self-assembly. This suggests that gastric organoids have the capacity to retain the spatial information and identity of the region of origin in a more complex system. Transcriptomic analyses revealed that native tissue samples, organoids and assembloids cluster separately, indicating differences between in vitro systems and native tissue. However, the MRA demonstrated a closer transcriptomic profile to the tissue cluster, compared with organoids alone. Our system showed overall increased levels of differentiation markers, confirmed at the transcriptomic and proteomic levels. Interestingly, the MRA showed expression of mature mucin-secreting and endocrine cells, but expressed a stem progenitor compartment at a comparable level to the tissue of origin. This contrasts with organoids, which retain a less differentiated transcriptomic profile.

In coherence with the increase in differentiation markers in MRAs, we observed a decrease in the expression of receptors that mediate a stem cell state (most notably LGR5). Of particular interest, we observed an increase in the expression of the ligand somatostatin, which was particularly expressed in the antral region of the MRA, with its receptor SSTR1 expressed over all of the regions of the MRA. Somatostatin is a peptide hormone, produced by δ cells, that plays a relevant role in controlling the function of parietal cells, thus regulating pH levels and maintaining the balance of digestive processes^38^. Of note, we observed that most of the ligands upregulated in MRAs were present in the antrum (that is, DLL4, FGF17, FGF5 and BMP2). Some of their corresponding receptors (NOTCH3, FGFR3, ERBB3, AREG and ACVR1) were also present in the other regions, suggesting a potential inter-region cross-talk that can only take place in vitro when the three areas are in contact, as in the MRA. Notably, the absence of co-culture in SRAs prevents these interactions from occurring. When comparing SRAs and MRAs, which are characterized by the same assembloid complexity and same ECM composition, the changes in pathways previously discussed could only be ascribed to the cross-talk between different gastric regions. In addition, a number of inflammation-related receptors were identified. Importantly, these assembloid structures have a considerably larger scale than single organoids. Consequently, there might be decreased medium diffusion in the inner part of the assembloid, or an accumulation of dead cells in the enclosed lumen^39^, impeding their elimination.

The generation of parietal cells in primary-stem-cell-derived gastric organoids has been challenging to date. We hypothesized that our MRA system could facilitate the emergence of this cell type. To identify the elusive parietal cell type, we adopted a single-cell proteomic approach^29–31^. Cytometry by time of flight confirmed some of the transcriptomic findings in the MRA. In particular, at the protein level, markers of stemness were reduced, alongside a reduction in proliferating stem cells, as indicated by the MRA cell-state profile. Concurrently, antral differentiation proteins such as gastrin and somatostatin increased in the antral region of the MRA. The main finding of the proteomic analysis was the presence in the MRA fundic region of the gastric-specific proton pump ATPase β, which is responsible for acid production. Overall, these results provide very interesting insights into gastric epithelial functionality. First, MRAs promote greater gastric differentiation compared with single-region organoids. Second, cross-communication between the regions is needed to promote this differentiation, as the duplets of single organoids that are cultured in the same Matrigel droplets but are not fused do not promote the same level of differentiation as MRAs. The presence of the active glycosylated form of ATPase H^+^/K^+^ transporting subunit β in MRAs, as found in parietal cells in vivo, was confirmed by WES analysis. However, this active form was not detected in SRA samples, confirming that cross-communication among regions is important for this process. Lastly, we further validated the presence of ATPase β-positive cells in MRAs through immunofluorescence, showing that their differentiation is affected by EGF receptor inhibition.

We were able to assess acid production in the MRA by live imaging with pH-reactive dyes. Functional assessment using live imaging confirmed the presence of active proton pumps and acid production in the fundic and body regions of the assembloid. Strikingly, the MRA responded to the administration of histamine, a nitrogenous compound that stimulates gastric acid secretion, and omeprazole, a drug that inhibits H^+^/K^+^ ATPase proton pumps, reducing gastric acid secretion. This did not occur when analysing SRAs under the same conditions.

Moreover, we demonstrated in vivo survival of the assembloid system upon transplantation in immunocompromised mice. Both SRAs and MRAs preserved the phenotypic characteristics of the assembloids with a larger lumen, as identified by iodinated microfocus computed tomography, and a complex epithelial wall with gastric mucous-secreting glands. The epithelium was able to maintain apicobasal polarity and a regionalized gastric identity.

Finally, using our MRA system we were able to produce a reliable patient-specific disease model. We provide clear evidence of defective N-glycosylation in the context of PMM2–HIPKD–IBD-associated *PMM2* variants in paediatric-patient-derived samples with a context- and cell-type-dependent effect. We also provide evidence of an impact on epithelial secretory function, which may be mechanistically linked to both the development of the unusual gastric pathological manifestations and the inflammatory intestinal pathology more broadly. The in vitro framework we have developed to model the gastrointestinal-specific effects of this disease offers a platform for preliminary testing of therapeutic agents targeting this specific pathway (for example, PMM2 activators under trial for the management of congenital disorder of glycosylation in patients with biallelic deleterious *PMM2* mutations)^40^.

Overall, our work shows the successful isolation of gastric organoids derived from different regions of the stomach and their capacity to self-organize into a complex multi-regional structure. These analyses demonstrate the occurrence of important gastric transcriptomic and protein changes upon self-assembly of the multi-organoid constructs. In particular, the floating culture system coupled with the multi-regional identity favours the functional maturation of differentiated cell types. We believe that our gastric MRAs can mimic complex in vivo stomach physiology, providing valuable insights into human gastric pathology and therapeutic discovery.

## Methods

### Ethics and licences

Human foetal stomachs were dissected from tissue obtained immediately after termination of pregnancy, in compliance with the bioethics legislation in the UK. Foetal samples were obtained from the joint Medical Research Council–Wellcome Trust Human Developmental Biology Resource under research tissue bank ethics approval from University College London (UCL) site Research Ethics Committee (reference: 18/LO/0822; Integrated Research Application System project ID: 244325). Samples were used for tissue characterization.

All paediatric gastric tissue was donated by the patient, or person with parental responsibility, after informed consent was obtained by an independent research coordinator at the relevant study site, under the approved research licence. Human gastric biopsies were collected and processed in compliance with the Human Tissue Act under licence 18DS02 by the National Health Service (NHS) Health Research Authority, East of England, Cambridge Central Research Ethics Committee. Paediatric samples were sourced from endoscopic mucosal biopsies at the Great Ormond Street Hospital (GOSH) NHS Foundation Trust. Samples were used to derive organoid lines or for native tissue characterization.

Animal care and procedures were in accordance with National Centre for the Replacement Refinement and Reduction of Animals in Research guidelines. Experimental work was undertaken under ethics approval governed by UK Home Office Project Licence PPL PDD3A088A and Personal Licence PIL I73E168C9.

### Gastric gland isolation

Gastric tissues were collected in ice-cold Advanced DMEM/F-12 medium (12634; Thermo Fisher Scientific) supplemented with 10 mM HEPES (15630080; Thermo Fisher Scientific), 2 mM GlutaMAX (35050061; Thermo Fisher Scientific) and 1% penicillin–streptomycin (15140122; Thermo Fisher Scientific), defined as ADMEM+++, and processed immediately following collection. A complete set of biopsies were isolated from each patient (endoscopically or by sample resection after gastrectomy), comprising fundus, body and antrum samples, which were processed separately. Glands were isolated using an established chelation–mechanical dissociation protocol^8,22^. Endoscopic gastric biopsies of a clean mucosal layer (3–5 mm diameter) were processed without further dissection. Tissues were washed in a petri dish with cold HBSS to remove debris, and a glass coverslip was then used to scrape away the luminal mucous layer. Tissues were then cut into smaller pieces (1 mm diameter) with a scalpel, transferred to a 15-ml tube containing 10 ml fresh HBSS and then washed vigorously using a 10-ml pipette pre-coated with 1% bovine serum albumin (BSA) (A9418; Sigma–Aldrich) in Dulbecco’s phosphate-buffered saline without calcium and magnesium ions (DPBS) (D8537; Sigma–Aldrich). After washing, the pieces were allowed to settle and the supernatant was discarded. This process was repeated until the supernatant was clear. The mucosal pieces were then incubated in a chelating buffer made up of 5.6 mmol l^−1^ Na_2_HPO_4_, 8.0 mmol l^−1^ KH_2_PO_4_, 96.2 mmol l^−1^ NaCl, 1.6 mmol l^−1^ KCl, 43.4 mmol l^−1^ sucrose, 54.9 mmol l^−1^ D-sorbitol, 0.5 mmol l^−1^ DL-dithiothreitol and 2 mM ethylenediaminetetraacetic acid (EDTA) (all from Sigma–Aldrich) in Milli-Q water (18.2 mW cm^−1^; Merck Millipore) for 30 min at 37 °C on a planar shaking platform. The chelating buffer was discarded and mucosal pieces were washed in ice-cold DPBS with calcium and magnesium ions (DPBS++; D8662; Sigma–Aldrich). Washed mucosal pieces were then transferred to a new 10-cm petri dish on ice and hand pressure was applied to the lid in contact with the mucosal surface with a 3.5-cm petri dish to release the gastric glands. The released glands were collected from the dish with ice-cold ADMEM+++ and filtered through a 40-μm cell strainer, then centrifuged at 200*g* for 5 min at 4 °C. The supernatant was aspirated and the glands were resuspended in ice-cold undiluted Matrigel Growth Factor Reduced (MGFR) (354230; Corning). Droplets of 30 μl were aliquoted into pre-warmed multi-well plates and incubated inverted for 20 min at 37 °C to induce gelation. Then, 10 μM of the Rho kinase inhibitor Y-27632 (1254; Tocris) was added to the medium for the first 3–4 days. Gastric organoid medium was added and changed every 3 days (Supplementary Table 2).

### Passaging of gastric organoids

For expansion, organoids were passaged by enzymatic single-cell dissociation. Organoids were retrieved from Matrigel with a ADMEM+++ wash while working on ice. After retrieval from the well and centrifugation at 300*g* for 5 min at 4 °C, the organoid pellet was resuspended in 1 ml TrypLE Express (12605010; Thermo Fisher Scientific), incubated at 37 °C for 5 min and manually disaggregated with a P1000. TrypLE was quenched with 10 ml ice-cold ADMEM+++ and centrifuged at 300*g* for 5 min at 4 °C. After aspirating the supernatant, single cells were then resuspended in MGRF at the desired split ratio (1:3 to 1:15) and plated in 30-μl droplets in pre-warmed multi-well tissue culture plates. Y-27632 was added to the medium for the first 24 h.

### Generation of reporter cell lines for single-cell experiments

Fundus–RFP and body–GFP reporter organoid lines were generated by electroporation based on previously described methods^41^. Briefly, 10 μM Y-27632 was added to the culture medium 2 d before electroporation and 1.25% dimethyl sulfoxide (DMSO) was added 1 day before electroporation. Organoids were dissociated to single cells with Accumax (00-4666-56; Thermo Fisher Scientific), filtered, centrifuged and resuspended in 1 ml Opti-MEM (31985062; Thermo Fisher Scientific) with Y-27632. PiggyBac vectors (1 μg ml^−1^) and piggyBac transposase (0.5 μg ml^−1^) were added to the cell suspension, transferred to a 2-mm electroporation cuvette and electroporated using an NEPA21 electroporator. Electroporated cells were transferred to a new 1.5-ml tube containing 400 ml Opti-MEM with 10 μM Y-27632 and centrifuged. The supernatant was then aspirated and the pellet resuspended in MGFR and plated. After MGFR polymerization, gastric medium with Y-27632 and 1.25% DMSO was added. DMSO was removed from the medium after 24 h. Organoids with successful integration of the respective plasmids were selected with puromycin at day 5 after electroporation.

### Design and fabrication of a custom culture well

A narrow linear culture well was designed to constrain the self-aggregation of organoids into a linear tube with fundus gastric organoids at one end and antrum gastric organoids at the other end. SOLIDWORKS 2020 (Dassault Systems) was used to design in 3D the well rectangular shape. The design was converted into coordinates using ideaMaker (Raise3D) to allow printing using an E2 desktop 3D printer (Raise3D). The mould was printed in polylactic acid using fused filament extrusion deposition. The layer height was set to 0.05 mm to optimize the smooth external surface of the walls of the mould. To complete the top of the pillars, an ironing function was applied, then the resulting structure was sanded by hand to smooth the surface. The resulting mould was used as a negative to cast a ten-well plate in PDMS. A pre-mixed 10:1 mixture of PDMS pre-polymer and curing agent solutions (SYLGARD 184 kit; Dow Corning) was cast and cured at 40 °C overnight.

### Production of collagen I hydrogel

The following methodology was adapted from a previous published protocol^42^. Rat tail collagen I was used to prepare a collagen I hydrogel of physiological pH and salinity using the recipe provided in Supplementary Table 3. Collagen I concentrations of 0.75, 2.00 and 4.50 mg ml^−1^ were tested, with 0.75 mg ml^−1^ proving optimal. This concentration was therefore used for all subsequent experiments. The collagen I hydrogel was prepared by mixing the components on ice with 1% BSA in DPBS-pre-coated wide-bore pipette tips. The pH of the hydrogel was tested with colorimetric pH strips and adjusted to pH 7.5, with 10 M sodium hydroxide (S5881; Sigma–Aldrich). The hydrogel was freshly prepared for each experiment and kept on ice until it was combined with the organoids.

### Floating gastric SRA and MRA cultures

On day 7 after seeding as single cells, gastric organoids were incubated in Cell Recovery Solution (354253; Corning) for 45 min on ice to dissolve the MGFR. Whole organoids were transferred to a 15-ml tube and washed twice in 10 ml ADMEM/F-12+++, then centrifuged at 200*g* for 5 min at 4 °C. Organoids were then resuspended in 200 μl collagen I pre-gel to be seeded and then plated in an ultra-low-attachment 24-well tissue culture plate (3473; Corning) in a ring shape for SRAs. For MRAs, the fundus, body and antrum organoids were seeded in the custom-designed plate. Specifically, two 30-μl drops of confluent fully grown organoids were used per region (to a total of total six drops per PDMS well). After being released from MGFR as described above, each region was encapsulated in 40 μl collagen I pre-gel (to a total of 120 μl per PDMS well). After pre-coating the PDMS wells with 1% BSA in DPBS, each region was loaded into a separate 200-μl pipette and delivered into the well simultaneously to recapitulate the fundus, body, antrum order of normal stomach. The plate was then incubated at 37 °C under 5% CO_2_ for 30 min to allow polymerization of the collagen–organoid. The gel structure was then detached from the bottom of the well by the forceful addition of 500 μl gastric organoid medium. The medium was changed every 1–2 days (depending on cell replication) until conclusion of the experiment at day 10.

When treated with erlotinib (10483-500-CAY; Cambridge Bioscience), the drug was added to the standard expansion medium at day 4 of the floating culture condition (20 nM) and maintained until the culture endpoint.

### Subcutaneous implantation of gastric assembloids in mice

Mice from Charles River Laboratories (12 weeks of age; non-obese diabetic, severe combined immunodeficient interleukin-2 receptor gamma null (NOD scid gamma (NSG); NOD.Cg-*Prkdc*^*scid*^*Il2rg*^*tm1Wjl*^/SzJ) were used in all of the in vivo implantation experiments. Mice were group housed in individually ventilated cages in a specialized immunodeficient colony room, with 12 h light/12 h dark cycles, at 20–24 °C and under 45–65% humidity, with food and water ad libitum. Following subcutaneous implantation procedures, mice were recovered individually in new individually ventilated cages before being returned to group housing with other mice that had undergone the same procedure. Access to standard diet and water was immediately provided following recovery. Each assembloid construct was loaded onto a disk of liquid MGFR contained in sterile silicone O-rings and allowed to polymerise for 15 min at 37 °C. MRA were orientated by application of a surgical clip to the fundus end of the tube prior to loading onto MGFR. After polymerization, the construct loaded ring was returned to gastric organoid complete medium at 37 °C and under 5% CO_2_ until the implantation procedure (<24 h). NSG mice were anaesthetized using 4% inhaled isoflurane in a sterile operating theatre within the UCL Institute of Child Health (ICH) Animal facility. The temperature was maintained using a heated operating table and mice were monitored intra-operatively by direct observation of the respiratory rate and pattern, as well as testing of the pedal reflex, allowing adjustment of the isoflurane concentration (maintenance concentration: 1.5–2.5%). A 1 cm × 1 cm area on the dorsum of the mouse was shaved and sterilized with dilute chlorhexidine before covering the mouse with a transparent sheet to aid sterility and thermoregulation. Bupivacaine (8 mg kg^−1^) was administered before making a 5-mm longitudinal incision under 2× optical magnification surgical loupes. Subcutaneous pockets were created by blunt dissection according to the number of samples being implanted (with a maximum of four per mouse). The wound was closed with interrupted subcuticular 5–0 VICRYL RAPIDE (polyglactin 910) sutures (VR493; Ethicon), and 0.1 mg kg^−1^ buprenorphine was administered subcutaneously for analgesia.

### Retrieval and processing of implanted constructs

Constructs were retrieved after 4 weeks of implantation. Mice were sacrificed by cervical dislocation and the constructs were dissected from the mouse and silicone O-ring under a Zeiss Discovery V20 SteREO microscope with a Zeiss CL 1500 HAL light source at 10–20× magnification. Images were acquired on the Zeiss microscope using a Zeiss Axiocam 506 colour camera. Samples for immunofluorescence were fixed in 4% paraformaldehyde (100496; Sigma–Aldrich) for 30 min before storage in DPBS with 1× antibiotic–antimycotic (15240096; Gibco) at 4 °C. For the molecular analyses, MRA constructs were divided with a scalpel into fundus, body and antral regions, with junctional zones discarded to avoid cross-region contamination, then minced and placed in RLT lysis buffer for RNA extraction.

### Immunofluorescence staining

Tissues, organoids and assembloids were fixed in 4% paraformaldehyde before processing. For tissue slides, blocking and permeabilization were performed using 0.5% Triton X-100 in 1% BSA for 2 h at room temperature (or 1 h of permeabilization in 0.5% Triton X-100 in PBS and 1 h of blocking in 10% FBS), then primary antibodies were incubated in permeabilization solution for 24 h at 4 °C. Primary antibodies were removed using three 2-h washes in 0.5% Triton X-100 at room temperature before overnight incubation of secondary antibodies at 4 °C. Finally, unbound secondary antibodies were washed three times in 2-h incubations of DPBS and the slides were then mounted for acquisition. For 3D organoid and assembloid whole-mount staining, fixed samples were quenched with 0.1 M NH_4_Cl for 60 min. After three further washes with DPBS, SRAs were cut into at least four pieces to facilitate multiple immunolabelling panels, whereas MRAs were labelled and subsequently imaged as intact structures. The benzyl alcohol and benzyl benzoate (BABB)-based clearing protocol was used^32^. Samples underwent dehydration in a methanol (34860; Sigma–Aldrich) series followed by incubation in 5% hydrogen peroxide in methanol for 2 h at 4 °C. Rehydration in reverse methanol series and equilibration in DPBS were performed before transfer of the samples to permeabilization solution (20% DMSO, 2.3% glycine (410225; Sigma–Aldrich) and 0.2% Triton X-100 in DPBS). Samples were permeabilized overnight at 4 °C, then transferred to blocking solution (10% DMSO, 6% normal donkey serum (D9663; Sigma–Aldrich) and 0.2% Triton X-100 in DPBS) for 24 h at room temperature. Primary antibodies were made up in 5% DMSO, 3% normal donkey serum, 0.2% Tween 20 (P1379; Sigma–Aldrich) and 0.1% heparin (H3149; Sigma–Aldrich) in DPBS and incubated with samples for 24 h at 4 °C. Primary antibodies were washed from the constructs with six 1-h washes in 0.2% Tween 20 and 0.1% heparin in DPBS. Secondary antibodies were prepared in the same solution as the primary antibodies and applied to the samples overnight at 4 °C. Secondary antibodies were removed by six 1-h washes in 0.2% Tween 20 and 0.1% heparin in DPBS until refractive index matching and imaging. All of the antibodies are listed in Supplementary Table 4.

### Acridine orange

Live MRAs were cut longitudinally in half. Cut samples were preincubated with or without omeprazole (O104; Sigma–Aldrich; 100 μM) for 1 h at 37 °C, according to the experimental condition. They were then incubated with 10 μM acridine orange (A1301; Thermo Fisher Scientific), with or without 100 μM histamine (H7127; Sigma–Aldrich) and 100 μM omeprazole. Images were acquired with a Zeiss Axio Observer A1 microscope.

Timelapse single-cell experiments were carried out by incubating with omeprazole (100 μM) for 1 h. Acridine orange was incubated for 15 min at 37 °C. Then, histamine was either added or not, this being the first time point of the experiment. Images were acquired in an automated way for 1 h (in a chamber at 37 °C) every 10 min (from *t*_0_ to *t*_5_) or every 20 min (*t*_0_ to *t*_3_), with 20-μm-thick Z slices for 3D reconstruction. Acquisition was performed by exiting acridine orange at *λ* = 488 nm and fluorescence information was collected at 600–650 nm (red) or 500–550 nm (green).

### Microscopy image acquisition

Brightfield images of organoids in culture were acquired using a Zeiss Axio Observer A1 inverted widefield microscope with a Colibri 5 LED light source and an AxioCam MRm camera system. Reporter cell lines were imaged in culture using the same system. Sectioned samples were acquired on either a Zeiss Axio Observer A1 microscope with a Colibri 7 LED light source and Hamamatsu Flash 4.0 V3 camera system or a Zeiss LSM 710 inverted single-photon confocal microscope. Widefield images were acquired using short Z stacks to allow later processing by deconvolution and Gaussian-based stack focusing for 2D projection. Whole-mount samples were acquired on the Zeiss LSM 710 confocal microscope on a FluoroDish.

Acridine orange image acquisition of whole regions at the final timepoint were acquired with a Zeiss Axio Observer A1 microscope with a Colibri 7 LED light source. Acridine orange timelapse imaging acquisition was performed with a Nikon Eclipse Ti2 inverted microscope.

### Image processing and analysis

Brightfield images were processed using Zeiss Zen Pro. Tissue sections were processed using batch deconvolution in Huygens Essential (Scientific Volume Imaging; Compute Engine 21.10.0p0.64b). Whole-mount-image datasets were imported into Fiji for processing. The colour and contrast were minimally adjusted. Stacks were represented as Z projections using maximum intensity projection incorporating all or selected slices, including single slices where this allowed optimal visualization of the data. Whole-mount-image datasets of SRAs and MRAs were exported from Zeiss Zen Pro to Imaris x64 (Bitplane; version 8.0.2; build 36053) for 3D and 3D stereo visualization using the Surpass 3D view. Rendering in 3D was done using transparent (maximum intensity projection) 3D mode and sections were performed with an OrthoSlicer and Clipping tools. Single- and multi-channel images were exported after optimization of the brightness and contrast in TIFF file format. Videos were made using the inbuilt Imaris point-to-point animation tool and then exported directly in MP4 file format.

### Transmission electron microscopy

Gastric assembloid samples were fixed in 2.5% glutaraldehyde in 0.1 M sodium cacodylate buffer, followed by secondary fixation in 1.0% osmium tetroxide. Tissues were dehydrated in graded ethanol, transferred to a transitional fluid (propylene oxide) and then infiltrated and embedded in Agar 100 epoxy resin. Polymerization was at 60 °C for 48 h. Then, 90-nm ultrathin sections were cut using a DiATOME diamond knife on a Leica Ultracut UC7 ultramicrotome. Sections were picked up on Athene 300 mesh copper grids and stained with 70% alcoholic uranyl acetate and Reynold’s lead citrate for contrast. The samples were examined using a JEOL 1400 transmission electron microscope and images were recorded using an AMT XR80 digital camera and software. Fundus, body and antrum regions were examined from each patient culture. Cells showing ultrastructural features were recorded at higher magnifications for further evaluation.

### Iodinated microfocus computed tomography

Implanted SRAs were iodinated by immersion overnight in 1.25% potassium triiodide (II0181; Chemondis) diluted 1:2 in 10% formalin (HT501128; Sigma–Aldrich). After washing with distilled water, samples were wrapped in laboratory film and mounted in nutrient agar (BO0336B; Thermo Fisher Scientific) in a 1.5-ml tube to separate it from the container during scanning. Micro-CT images of the specimen were acquired with a MedX mCT scanner (Nikon Metrology) using a molybdenum target. The X-ray energy was 90 kV, with a beam current of 89 μA. The exposure time was 708 ms, at four frames per projection, with the number of projections optimized for the size of the specimen. The resulting total average scan time was 2 h with a voxel size of 4.33 µm. Projection images were reconstructed with modified Feldkamp filtered back-projection algorithms with proprietary software (CTPro3D; Nikon Metrology) and processed afterwards with VGSTUDIO MAX (Volume Graphics; version 3.2).

### Library preparation, sequencing, alignment and initial processing

RNA extraction was performed using an RNeasy Mini Kit (Qiagen) following the manufacturer’s instructions. For library preparation, total RNA quantification and integrity were confirmed using Agilent’s 4200 TapeStation (Standard Total RNA assay). For each sample, 10 ng total RNA was processed using the KAPA mRNA HyperPrep Kit (KK8580; Roche) for patient 1 and the NEBNext Low Input RNA Library Prep Kit (E6420; New England Biolabs) for patients 2, 3 and 4, according to the manufacturer’s instructions. For sequencing, high-yield, adaptor–dimer free libraries were confirmed on an Agilent TapeStation 4200 (Agilent High Sensitivity DNA 1000 assay) and a library quantification was estimated using the Qubit dsDNA HS assay (Life Technologies). Libraries were normalized to 4 nM and then equal volumes of each were pooled together. The library pool was denatured and sequenced on a NextSeq 2000 (patient 1) or NovaSeq 6000 (patients 2, 3 and 4) (both Illumina) at 750 pM, with a 56-bp paired-end read for patient 1 and a 101-bp single-end read for patients 2, 3 and 4, run with corresponding 8-bp unique dual-sample indexes and an 8-bp unique molecular index. For data analysis, run data were demultiplexed and converted to fastq files using Illumina’s BCL Convert Software (version 3.7.5).

### RNA-seq analysis

The identified genes were filtered out if not expressed at a count per million (CPM) higher than one in at least two samples of at least one condition. Data were normalized using the trimmed mean of *M* values normalization method implemented in the edgeR package (version 4.2.1)^43^. A PCA was performed using the R function prcomp on log_2_[CPM + 1] data, after centring. Data visualization was performed with the R package ggplot2 version 3.5.1 (ref. ^44^). DEGs were computed with edgeR using mixed criteria based on a *P* value (after false discovery rate correction with the Benjamini–Hochberg method) of <0.01 and an absolute fold change of >2, unless otherwise specified. Hierarchical clustering and heat map visualization were performed using the R package pheatmap (version 1.0.12) with Euclidean distance and complete linkage. Functional enrichment analysis of Reactome pathways (updated in 2022) was performed by right-sided hypergeometric test using ClueGO (version 2.5.10)^45^ and CluePedia (version 1.5.10)^46^ within the Cytoscape version 3.10.0 environment^47^.

Due to the different sequencing procedure described above, data corresponding to patients 2, 3 and 4 were analysed separately from data from patient 1. The analysis was performed according to the same methods above, except for the addition of batch effect correction to take into account the two different rounds of sequencing, using the combat_seq function^48^ embedded in the sva package (version 3.52.0)^49^. Moreover, the DEG analysis for patients 2, 3 and 4 included patient number as a factor in the model.

Proteins playing a role as ligands or receptors were selected according to Ramilowski et al.^50^. Receptor genes were identified as being differentially expressed in comparisons of MRAs versus organoids (Supplementary Data 1 and 2) and MRAs versus SRAs (Supplementary Data 3 and 4) within the same sub-tissue, whereas ligand genes were identified as being upregulated if differentially expressed in any MRA sub-tissue versus organoid- or SRA-specific sub-tissue (tissues were matched to those used to determine differentially expressed receptor genes for receptor–ligand pair analysis). Chord diagrams were generated using the chordDiagram function embedded in circlize package (version 0.4.16)^51^. RNA-seq data are publicly available from the Gene Expression Omnibus repository with accession numbers GSE247115 and GSE280843.

### TOBis MC analysis

For cell dissociation, we optimized and adapted the published TOBis protocol^28,29^. Before barcoding, pre-treated and fixed MRA-containing RFP and GFP reporter cell lines were cut with a scalpel into several pieces. Each piece was assigned a different barcode to preserve the spatial orientation in the analysed samples. Control organoids in MGFR culture were assigned separate barcodes. Barcodes were premade in the laboratory of C.J.T. and applied to the samples overnight at 4 °C in rotation. Unbound barcodes were quenched using 1 mM glutathione (G6529; Sigma–Aldrich) in Maxpar Cell Staining Buffer (CSB) (201068; Standard BioTools). Dissociation solution was prepared fresh for each experiment, comprising 0.5 mg ml^−1^ dispase II (17105041; Thermo Fisher Scientific), 1 mg ml^−1^ collagenase IV (17104019; Thermo Fisher Scientific) and 0.2 mg ml^−1^ DNase I (DN25; Sigma–Aldrich) final working concentrations in phosphate-buffered saline (PBS). Samples were placed in gentleMACS C Tubes (130-093237; Miltenyi Biotec) in 5 ml dissociation solution and run on a gentleMACS Octo Dissociator (Miltenyi Biotec) at 37 °C in 15-min cycles for three to five cycles during the optimization phase. Each cycle comprised forward spin at 50 rpm for 1 min, backward spin at 50 rpm for 1 min, forward spin at 1,500 rpm for 2 s, backward spin at 1,500 rpm for 2 s and forward spin at 100 rpm for 1 min (looped ×10). After dissociation, the samples were centrifuged, washed twice in CSB and filtered through a 40-µm polyethylene terephthalate filter. A sample of filtered suspension was stained with Trypan Blue (15250061; Thermo Fisher Scientific), counted in a Countess Automated Cell Counter (Thermo Fisher Scientific) and inspected on a brightfield microscope for cell morphology and the presence of doublets and undigested ECM. For staining using rare-Earth-metal-conjugated antibodies, antibodies for cell type were selected from an optimized panel. BSA- and azide-free formulations of the antibodies, using the same clone as in immunofluorescence where available, were custom conjugated to rare Earth metals using Maxpar X8 polymers (201300; Standard BioTools). The antibodies used in the TOBis MC experiments are shown in Supplementary Table 5. Antibodies against extracellular epitopes were made up in 50 µl CSB at optimized concentrations and incubated with cells for 30 min at room temperature on a rocker. After washing with CSB, cells were permeabilized with 0.1% Triton X-100 in PBS for 30 min at room temperature on a rocker, washed with CSB again, further permeabilized with 50% methanol in PBS for 10 min on ice and then washed with CSB. Antibodies against intracellular epitopes were made up in 50 µl CSB and applied to the cells for 30 min at room temperature on a rocker. Next, stained cells were re-fixed with 1.6% formaldehyde in PBS (28906; Pierce) for 10 min at room temperature. After washing with CSB, DNA intercalation was performed using 125 nM Cell-ID Intercalator-Ir (201192A; Standard BioTools) in Maxpar Fix and Perm Buffer (201067; Standard BioTools) overnight at 4 °C, in which cells were stored until they were run on the CyTOF XT (Standard BioTools). For mass cytometry data acquisition, stained cells were washed with CSB and then resuspended in Maxpar CAS+ solution (201244; Standard BioTools) at ~0.5–1.0 × 10^6^ cells per ml. EQ6 beads (201245; Standard BioTools) were added at a volumetric ratio of 1:5 to the cell suspension, and EDTA was added to a final concentration of 2 mM. Cells were loaded into the CyTOF XT and events were acquired using Standard BioTools CyTOF software, aiming for 100–400 events per second. Raw data were processed using Standard BioTools CyTOF software version 6.7 to remove EQ beads and normalize signals before exporting the data as FCS files.

After acquisition, the processed file was debarcoded (https://github.com/zunderlab/single-cell-debarcoder) and uploaded onto the Cytobank platform for further analysis. Single cells were gated for Gaussian parameters, cisplatin^low^ and DNA^high^ to remove debris, doublets and any other non-cellular-resembling matter from the data. Uniform manifold approximation and projection analysis was performed using the default settings in Cytobank (https://www.cytobank.org/). The individual debarcoded files were downloaded from Cytobank as text files and pre-processed using the mass cytometry data analysis pipeline CyGNAL (https://github.com/TAPE-Lab/CyGNAL), then the Earth mover’s distance scores were computed. Once computed, heatmaps and PCA plots were generated and compiled using OmniGraffle professional.

### WES analysis

Protein extracts were prepared in RIPA buffer (R0278; Sigma–Aldrich) with protease inhibitor 1X (ab270055; Abcam) and quantified using a Pierce BCA Protein Assay Kit (23227; Thermo Fisher Scientific). Protein extracts were diluted at 0.3 mg ml^−1^ with Antibody Diluent 2 (042-203; Bio-Techne) and used for WES analysis, using a Wes machine (004-600; Bio-Techne). A 12–230 kDa separation module (004-600; Bio-Techne) was used, combined with the primary antibody H^+^/K^+^ ATPase β (MA3-923; Thermo Fisher Scientific), used at 1:25 dilution, and the anti-mouse detection module (DM-002; Bio-Techne). Sample normalization was done using the total protein detection module for chemiluminescence (DM-TP01; Bio-Techne). The results were visualized using the Compass for Simple Western software (version 6.1.0).

### Real-time PCR

RNA reverse transcription was performed using a High-Capacity cDNA Reverse Transcription Kit (Thermo Fisher Scientific) according to manufacturer’s instructions. Reverse transcription was done using a T100 Thermal Cycler (Bio-Rad). The qRT-PCR was performed with custom-designed Thermo Fisher probes (Supplementary Table 6) using Universal SYBR Green Master Rox (4913850001; Merck Life Sciences) according to the manufacturer’s instructions. Reactions were performed on a QuantStudio 5 system (Thermo Fisher Scientific) and the results were analysed with QuantStudio Design & Analysis software (version 1.5.2; Thermo Fisher Scientific). Glyceraldehyde-3-phosphate dehydrogenase expression was used to normalize the Ct values for gene expression, and the data are shown as the relative fold change compared with controls (antrum organoids), using the ΔΔCt method, and presented as means ± s.e.m. GraphPad Prism Mac (version 10.3.0) was used to produce the graphs and for statistical analysis.

### Statistics and reproducibility

The statistical significance of reported results was assessed using statistical tests during data analyses, as indicated in the figure captions. Tissue samples were stained from two different patients, by two independent operators (Fig. 1b and Extended Data Fig. 1a,b). Cell culture experiments were replicated within the laboratory by at least three independent operators, with a minimum of *n* = 3 repeats per experiment. The assembloid formation protocol was optimized by four different operators, using more than ten organoids and collagen concentrations. Final optimized settings were used to perform all of the following experiments in the manuscript (Extended Data Fig. 2). Immunofluorescence stainings of organoids, MRAs, tissue and MRA slices were performed more than three times by two independent operators (Figs. 1f, 2b–i and 4d and Extended Data Figs. 2e,f and 8a,b). WES analyses were performed *n* = 3 times by one operator. Functional assays (acridine orange) were repeated *n* = 3 times by one operator, with blind analysis performed by another operator (Figs. 4c and 5k and Extended Data Fig. 8c). Hyperplastic-like budding formations were assessed *n* = 3 times by immunostaining and *n* = 1 time by haematoxylin and eosin (Fig. 5e,f). Mucin organoid staining was performed *n* = 4 times (Fig. 5h). GFP/RFP organoid line labelling was performed once (Extended Data Fig. 6). The mouse grafting experiments were repeated twice by two different operators. The sequencing analysis was performed once on each sample, as reported. The TOBis analysis was performed once after a first pilot experimental setup with three technical replicates per condition.

All attempts at replication were successful.

### Reporting summary

Further information on research design is available in the Nature Portfolio Reporting Summary linked to this article.

## Supplementary information

Supplementary Information Supplementary Tables 1–6.

Reporting Summary

Supplementary Datasets Supplementary Dataset 1. DEG analysis results (MRA versus organoids; patient 1). Supplementary Dataset 2. DEG analysis results (MRA versus organoids; patients 2, 3 and 4). Supplementary Dataset 3. DEG analysis results (SRA versus MRA; patient 1). Supplementary Dataset 4. DEG analysis results (SRA versus MRA; patients 2, 3 and 4).

Supplementary Video 1 **Collagen I hydrogel contraction test** Contrast phase timelapse of SRA formation at different concertation of collagen I hydrogel in a 24 well plate. Collagen I concentrations: 0.75 mg.mL (left), 2 mg/mL (centre), 4.5 mg/mL right.

Supplementary Video 2 **MRA immunostaining** 3D reconstruction of whole mount immunofluorescence of MRA. Nuclei in blue, Somatostatin in green, Chromogranin A in red, and PDX1 in yellow. Scale bar reported on each frame. 3D reconstruction with IMARIS.

Supplementary Video 3 **MRA-antral side immunostaining** 3D reconstruction of whole mount immunofluorescence of the antral side of MRA. Nuclei in blue, Somatostatin in green, Chromogranin A in red, and PDX1 in yellow. Scale bar reported on each frame. 3D reconstruction with IMARIS

Supplementary Video 4 **Acridine orange – Control** Timelapse of Acridine orange staining of Fundus, Body, and Antrum in MRA Control condition. Images acquired every 10 mins.

Supplementary Video 5 **Acridine orange – Histamine treatment** Timelapse frames of Acridine orange staining of Fundus, Body, and Antrum in MRA Histamine treatment (100 μM) condition. Images acquired every 10 mins.

Supplementary Video 6 **Acridine orange – Histamine + Omeprazole treatment** Timelapse frames of Acridine orange staining of Fundus, Body, and Antrum in MRA Histamine (100 μM) + Omeprazole (100 μM) condition. Images acquired every 10 mins.

Supplementary Video 7 **Microfocus computed tomography (Micro-CT)** Microfocus computed tomography (Micro-CT) of transplanted SRA overview.

Supplementary Video 8 **Microfocus computed tomography (Micro-CT)** Microfocus computed tomography (Micro-CT) of transplanted SRA axial and coronal views.

## Source data

Source Data Figs. 1, 3, 4 and 5 and Extended Data Figs. 1, 3, 7, 8, 9 and 10 Raw data used for graph preparation.

## Data Availability

All of the data supporting the findings of this study are available within the Article, its Supplementary Information, Source Data files and online deposited data. The gastric RNA-seq data generated in this study have been deposited in the National Center for Biotechnology Information Gene Expression Omnibus database with the accession codes GSE247115 and GSE280843. The TOBis MC data are deposited at https://community.cytobank.org/cytobank/experiments/113293. The Reactome Pathway Database (https://reactome.org) was used for functional enrichment analysis. Source data are provided with this paper.
