# Supplementary material for: Human gastric multi-regional assembloids for functional parietal maturation and patient-specific modelling of antral foveolar hyperplasia
Source: Nat Biomed Eng. 2026 Jan 23;10(10):2059–76. doi: 10.1038/s41551-025-01553-y (PMC13672061; doi:10.1038/s41551-025-01553-y)
Supplement: Supplementary file 1 [file 41551_2025_1553_MOESM1_ESM.pdf]

nature biomedical engineering

Supplementary information

https://doi.org/10.1038/s41551-025-01553-y

# Human gastric multi-regional assembloids for functional parietal maturation and patient-specific modelling of antral foveolar hyperplasia

In the format provided by the authors and unedited

## Supplementary Tables

**Supplementary Table 1.** Gastric samples list.

| Sample | Stage | Age | Region | Use |
|---|---|---|---|---|
| 1 | Early fetal | PCW 10 | Whole | Tissue Immunostaining |
| 2 | Pediatric | 4 yo | Antrum | Tissue Immunostaining, Expansion, Sequencing, Organoids Immunostaining, Assembloid generation, Assembloid Immunostaining, TOBis, In vivo implantation |
| 3 | Pediatric | 4 yo | Body | Tissue Immunostaining, Expansion, Sequencing, Organoids Immunostaining, Assembloid generation, Assembloid Immunostaining, In vivo implantation |
| 4 | Pediatric | 4 yo | Body_GFP | Expansion, Assembloid generation, TOBis, In vivo implantation, Sequencing |
| 5 | Pediatric | 4 yo | Fundus | Tissue Immunostaining, Expansion, Sequencing, Organoids Immunostaining, Assembloid generation, Assembloid Immunostaining, In vivo implantation |
| 6 | Pediatric | 4 yo | Fundus_FRP | Expansion, Assembloid generation, TOBis, In vivo implantation, Sequencing |
| 7 | Pediatric | 7 yo | Antrum | Tissue Immunostaining, Expansion, Organoids immunostaining, Assembloid generation, WES analysis, Sequencing, RT-qPCR |
| 8 | Pediatric | 7 yo | Body | Tissue Immunostaining, Expansion, Organoids immunostaining, Assembloid generation, WES analysis, Sequencing, RT-qPCR |
| 9 | Pediatric | 7 yo | Fundus | Tissue Immunostaining, Expansion, Organoids immunostaining, Assembloid generation, WES analysis, Sequencing, RT-qPCR |
| 10 | Pediatric | 11 yo | Antrum | Expansion, Organoid immunostaining, Assembloid generation, WES analysis, AO staining, TEM analysis, Sequencing, RT-qPCR |
| 11 | Pediatric | 11 yo | Body | Expansion, Organoid immunostaining, Assembloid generation, WES analysis, AO staining, TEM analysis, Sequencing, RT-qPCR |
| 12 | Pediatric | 11 yo | Fundus | Expansion, Organoid immunostaining, Assembloid generation, WES analysis, AO staining, TEM analysis, Sequencing, RT-qPCR |
| 13 | Pediatric | 6mo | Antrum | Expansion, Organoid immunostaining, Assembloid generation, WES analysis, AO staining, Sequencing, RT-qPCR |
| 14 | Pediatric | 6mo | Body | Expansion, Organoid immunostaining, Assembloid generation, WES analysis, AO staining, Sequencing, RT-qPCR |
| 15 | Pediatric | 6mo | Fundus | Expansion, Organoid immunostaining, Assembloid generation, WES analysis, AO staining, Sequencing, RT-qPCR |
| 16 | Pediatric | 8yo | Antrum | Expansion, Organoid immunostaining, Assembloid generation, WES analysis, AO staining, RT-qPCR, TEM analysis |
| 17 | Pediatric | 8yo | Body | Expansion, Organoid immunostaining, Assembloid generation, WES analysis, AO staining, RT-qPCR, TEM analysis |
| 18 | Pediatric | 8yo | Fundus | Expansion, Organoid immunostaining, Assembloid generation, WES analysis, AO staining, RT-qPCR, TEM analysis |
| 19 | Pediatric | 8yo | Hyperplastic region | Expansion, Organoid immunostaining, Assembloid generation, WES analysis, AO staining, RT-qPCR, TEM analysis |
| 20 | Pediatric | 8yo | Antrum | Expansion, Organoid immunostaining, Assembloid generation, WES analysis, AO staining, RT-qPCR, TEM analysis |
| 21 | Pediatric | 8yo | Body | Expansion, Organoid immunostaining, Assembloid generation, WES analysis, AO staining, RT-qPCR TEM analysis |
| 22 | Pediatric | 8yo | Fundus | Expansion, Organoid immunostaining, Assembloid generation, WES analysis, AO staining, RT-qPCR TEM analysis |
| 23 | Pediatric | 14yo | Antrum | Expansion, Organoid immunostaining Assembloid generation |
| 24 | Pediatric | 14yo | Body | Expansion, Organoid immunostaining Assembloid generation |
| 25 | Pediatric | 16yo | Antrum | Expansion, Organoid immunostaining Assembloid generation |
| 26 | Pediatric | 16yo | Body | Expansion, Organoid immunostaining Assembloid generation |
| 27 | Pediatric | 16yo | Fundus | Expansion, Organoid immunostaining Assembloid generation |

**Supplementary Table 2.** Gastric organoids medium composition

| Component | Stock conc. | Final conc. |
|---|---|---|
| Advanced DMEM F-12 (Thermo 12634) | - | To volume |
| HEPES (Thermo 15630080) | 1 M | 10 mM |
| Glutamax (Thermo 35050061) | 100 X | 2 mM |
| Pen/Strep (Thermo 15140122) | 100 % | 1 % |
| Primocin (Thermo Fisher NC9392943) | 50 mg/mL | 100 μg/mL |
| B-27 supplement minus vitamin A (Thermo 12587010) | 50 X | 1 X |
| n-acetylcysteine (Sigma A9165) | 500 mM | 1.25 mM |
| Wnt-3A (Peprotech 315-20) | 50 μg/mL | 100 ng/mL |
| R-spondin 1 (Peprotech 120-38) | 100 μg/mL | 500 ng/mL |
| Noggin (Peprotech 120-10C) | 100 μg/mL | 100 ng/mL |
| Human EGF (Thermo Fisher PMG8043) | 500 μg/mL | 50 ng/mL |
| Gastrin (Sigma G9020) | 100 μM | 10 nM |
| GSK-3 inhibitor (CHIR 99021) (Tocris 4423) | 3 mM | 3 μM |
| TGFβ inhibitor (A83-01) (Sigma SML0788) | 500 μM | 5 μM |
| FGF10 (Peprotech 100-26) | 100 μg/mL | 200 ng/mL |
| ROCK inhibitor Y-27632 (Tocris 1254) | 10 mg/mL | 10 μg/mL |

**Supplementary Table 3.** Collagen I Hydrogel composition (0.75mg/mL)

| Reagent | Company | Catalogue | Stock Concentration | Final Concentration |
|---|---|---|---|---|
| MilliQ water (18.2mΩ/cm) | Merck Millipore | ZIQ7003T0 | - | To volume |
| Advanced DMEM/F-12 | Thermo Fisher Scientific | 12500062 | 10X | 1X |
| HEPES | Thermo Fisher Scientific | 15630080 | 1 M | 10 mM |
| Collagen I (rat tail) | First Link | 60-35-810 Batch: RTC8328 | 5.05 mg/mL | 0.75 mg/mL |

**Supplementary Table 4.** Immumostaining Antibody List

| Antibody | Code | Brand | Host | Dilution |
|---|---|---|---|---|
| ATPase H+/K+ transporting subunit beta (ATP4B) | MA3-923 | Thermo | Mouse | 1:100 |
| Chromogranin A (CHGA) | ab15160 | Abcam | Rabbit | 1:50 |
| E-cadherin (E-CAD) | 610181 | BD bioscience | Mouse | 1:100 |
| Gastrin (GAST) | ab223501 | Abcam | Rabbit | 1:100 |
| Ghrelin (GHRL) | NB600-813 | Novus Biology | Goat | 1:100 |
| Integrin beta-4 (INTB4) | ab110167 | Abcam | Rat | 1:200 |
| Iroquois Homeobox 3 (IRX3) | Sc-166657 | Santa Cruz | Mouse | 1:100 |
| Lysozyme (LYZ) | GTX72913 | GeneTex | Rabbit | 1:100 |
| Mucin 5AC (MUC5AC) | ma5-12178 | Invitrogen | Mouse | 1:100 |
| Mucin 6 (MUC6) | ab216017 | Abcam | Mouse | 1:100 |
| Pancreatic and duodenal homeobox 1 (PDX1) | AF2419 | R&D SYSTEMS | Goat | 1:100 |
| Pepsinogen (PGC) | HPA031717 | Atlas | Rabbit | 1:100 |
| Somatostatin (SST) | MAB2358 | R&D Systems | Rat | 1:100 |
| | | | | |
| anti-Mouse 488 (secondary AB) | A11001 | Thermo Fisher | Goat | 1:300 |
| anti-Mouse 568 (secondary AB) | A10037 | Thermo Fisher | Goat | 1:300 |
| anti-Mouse 647 (secondary AB) | A31571 | Thermo Fisher | Donkey | 1:300 |
| anti-Rabbit 568 (secondary AB) | A11042 | Thermo Fisher | Donkey | 1:300 |
| Anti-Rat 488 (secondary AB) | A48269 | Thermo Fisher | Donkey | 1:300 |
| Anti-rabbit 647 (secondary AB) | A-21244 | Thermo Fisher | Goat | 1:300 |
| Anti-Goat 633 (secondary AB) | A-21082 | Thermo Fisher | Donkey | 1:300 |
| anti-Rat 594 (secondary AB) | A11007 | Thermo Fisher | Goat | 1:300 |
| Phalloidin 647 | A22287 | Thermo Fisher | --- | 1:500 |
| Hoechst 33342 (nuclear staining) | H1399 | Thermo Fisher | --- | 10 μg/mL |

**Supplementary Table 5.** TOB*is* MC Antibody List

| Antibody | Code | Brand | Metal |
|---|---|---|---|
| RFP | 200-301-379 | eBiosciences | Cd |
| mCHERRY | M11217 | Thermofisher | Cd |
| IdU | Q-27194 | Fluidigm | IdU |
| cCaspase3 [D175] (v4) | #9661 | CST | Nd |
| Stathmin1 | NBP1-76798 | Bio-techne | Nd |
| pNDRG1 [T346] (v2) | #5482 | CST | Nd |
| Lysozyme | 5490-4110 | Biorad | Nd |
| pSRC [Y418] (v8) | 50-9034-42 | eBioscience | Nd |
| p4E-BP1 [T37/46 ] (v3) | #2855 | CST | Sm |
| pRB [S807/811] (v5) | 558389 | BD Biosciences | Nd |
| pAKT [T308] (v9) | 558275 | BD Biosciences | Sm |
| pCREB [S133] (v11) | #9198 | CST | Eu |
| SOX9 (v3) | Ab76997 | Abcam | Yb |
| pMKK3/MKK6 [S189/207] (v4) | #9231 | CST | Gd |
| pP38 [T180/Y182] (v4) | #9211 | CST | Gd |
| GHRL1 | H00051738-M01 | Abcam | Ho |
| Gastrin | ab223501 | Abcam | Dy |
| LRIG1 (V3) | AF7498 | R&D Syustems | Dy |
| Pepsinogen C | ab255826 | Abcam | Yb |
| pP120-Catenin [T310] (V2) | 558203 | BD Bioscinces | Dy |
| Mucin5AC | MA5-12178 | Abcam | Er |
| pGSK3b [S9] (S2) | #9336 | CST | Er |
| Mucin6 | ab216017 | Abcam | Er |
| pSMAD2 [S465/467] /pSMAD3 [S423/425] (v5) | #8828 | CST | Er |
| CHGA | sc-393941 | Santa Cruz | Er |
| Somatostatin | MAB2358 | Atlas | Yb |
| cPARP [D214] (2) (v3) | #5626 | CST | Yb |
| K-ATPase_ATP4B | Ab176992 | Abcam | Lu |
| Cyclin B1 | 554179 | BD Biosciences | Yb |
| pHistone H3 [S28] | 641010 | BioLegend | Y |
| PDX1 | AF2419 | R&D Systems | Yb |
| GFP (V2) | 50-6498-82 | eBiosciences | Cd |
| LGR5 | MAB82401 | R&D Systems | Sm |

**Supplementary Table 6.** qRT-CPR primer list

| Gene | Forward | Rerverse |
| --- | --- | --- |
| *GAPDH* | CACATCGCTCAGACACCA TG | TGACGGTGCCATGGAATTT G |
| *MUC5AC* | GCTTTCTTGTGGAGGGTGTG | GATCACCATGTCCAAGCGTC |
| *ATP4B* | GACCTTCAACAATCCCCACG | TTCTCTGGAGTTCGCACACT |
| *PDX1* | TCCTACAGCACTCCACCTTG | ACTGGCATCAATTTCACGGG |
| *NOTCH1* | CAACTGCCAGAACCTTGTGC | GGCAACGTCAACACCTTGTC |
| *NOTCH3* | CTGGAGATTGACAACCGGCT | ACGAGAATGACCAGCAGCAA |
| *DLL4* | CCCCATGCCTCCAACTACTG | TTCCAACACACTGCCCTGAG |
| *FGFR4* | GCAGCCCAACACAGAGTACT | AGAACCACCTGACAAGCGAG |
| *FGF11* | AAAACGTGGAGTGCTAGGGG | GTTGGGTGGAGACTGAGTGG |
