# Supplementary material for: Human gastric multi-regional assembloids for functional parietal maturation and patient-specific modelling of antral foveolar hyperplasia
Source: Nat Biomed Eng. 2026 Jan 23;10(10):2059–76. doi: 10.1038/s41551-025-01553-y (PMC13672061; doi:10.1038/s41551-025-01553-y)
Supplement: Supplementary file 2 [file 41551_2025_1553_MOESM2_ESM.pdf]

nature portfolio

Corresponding author(s): Paolo De Coppi
Giovanni Giuseppe Giobbe

Last updated by author(s): May 15, 2025

# Reporting Summary

Nature Portfolio wishes to improve the reproducibility of the work that we publish. This form provides structure for consistency and transparency in reporting. For further information on Nature Portfolio policies, see our Editorial Policies and the Editorial Policy Checklist.

## Statistics

For all statistical analyses, confirm that the following items are present in the figure legend, table legend, main text, or Methods section.

| n/a | Confirmed | |
|---|---|---|
| ☐ | ☒ | The exact sample size ($n$) for each experimental group/condition, given as a discrete number and unit of measurement |
| ☐ | ☒ | A statement on whether measurements were taken from distinct samples or whether the same sample was measured repeatedly |
| ☐ | ☒ | The statistical test(s) used AND whether they are one- or two-sided<br>*Only common tests should be described solely by name; describe more complex techniques in the Methods section.* |
| ☒ | ☐ | A description of all covariates tested |
| ☐ | ☒ | A description of any assumptions or corrections, such as tests of normality and adjustment for multiple comparisons |
| ☐ | ☒ | A full description of the statistical parameters including central tendency (e.g. means) or other basic estimates (e.g. regression coefficient) AND variation (e.g. standard deviation) or associated estimates of uncertainty (e.g. confidence intervals) |
| ☐ | ☒ | For null hypothesis testing, the test statistic (e.g. $F$, $t$, $r$) with confidence intervals, effect sizes, degrees of freedom and $P$ value noted<br>*Give P values as exact values whenever suitable.* |
| ☒ | ☐ | For Bayesian analysis, information on the choice of priors and Markov chain Monte Carlo settings |
| ☒ | ☐ | For hierarchical and complex designs, identification of the appropriate level for tests and full reporting of outcomes |
| ☒ | ☐ | Estimates of effect sizes (e.g. Cohen's $d$, Pearson's $r$), indicating how they were calculated |

*Our web collection on statistics for biologists contains articles on many of the points above.*

## Software and code

Policy information about availability of computer code

**Data collection**

Image acquisition: Brightfield images of organoids in culture were acquired using on a Zeiss Axio Observer A1 inverted widefield microscope with a Colibri 5 LED light source and Axiocam MRm camera system. Reporter cell lines were imaged in culture using the same system. Sectioned samples were acquired on either a Zeiss Axio Observer A1 microscope with a Colibri 7 LED light source and Hamamatsu Flash 4v3 camera system or on a Zeiss LSM 710 inverted single photon confocal microscope. Widefield images were acquired using short Z-stacks to allow later processing by deconvolution and Gaussian-based stack focusing for 2D projection. Whole mount samples were acquired on the Zeiss LSM 710 confocal microscope on a FluoroDish. Acridine orange live imaging acquisition was performed with Nikon Eclipse Ti2 inverted microscope. For TEM images, the samples were examined in a JEOL 1400 transmission electron microscope and images recorded using AMT XR80 digital camera and software. Micro-CT images of the specimen were acquired with a MedX mCT scanner (Nikon Metrology) using a molybdenum target.

RNA sequencing: The library pool was denatured and sequenced on the NextSeq 2000 instrument (Illumina, San Diego, US) at 750pM, using a 56bp paired-read run with corresponding 8bp unique dual sample indexes and 8bp unique molecular index. For data analysis, run data were demultiplexed and converted to fastq files using Illumina's BCL Convert Software v3.7.5.

Thiol organoid barcoding in situ mass cytometry (TOBis MC): For mass cytometry data acquisition, stained cells were washed with CSB and then resuspended in MaxPar CAS+ solution (StandardBioTools, 201244) at ~0.5-1.0 x 106 cells per mL. EQ6 beads (StandardBioTools, 201245) were added at 1:5 volumetric ratio and EDTA to a final concentration of 2 mM. Cells were loaded into the CyTOF XT� and events were acquired using StandardBioTools CyTOF� software aiming for 100-400 events per second

WES (Simple Western™ Automated Western Blot Systems): Protein extracts were prepared in RIPA buffer (R0278, Sigma) with protease inhibitor 1X (ab270055, Abcam) and quantified with Pierce BCA Protein Assay Kit (23227, ThermoFisher). Protein extracts were diluted at

0.3mg/mL with Antibody diluent 2 (042-203, Bio-techne) and used for Simple Western analysis, using Wes machine (004-600, Bio-techne).

RNA reverse transcription was performed using the High-Capacity cDNA Reverse Transcription Kit (Thermo Fisher), according to manifacturer's instructions. Reverse transcription was done using the T100 thermal cycler (Bio-Rad) the qRT-PCR was performed with custom designed Thermo Fisher probes using Universal Sybr Green Master Rox (Merck Life Sciences, 4913850001) according to manifaturr's instructions. Reactions were performed on QuantStudio� 5 (Thermo Fisher).

Data analysis

Image processing and analysis: Brightfield images were processed using Zeiss Zen Pro. Tissue sections were processed using batch deconvolution in Huygens Essential (Scientific Volume Imaging, compute engine 21.10.0p0.64b). Whole mount image datasets were imported to Fiji for processing. Colour and contrast were minimally adjusted. Stacks were represented as Z-projections using maximum intensity projection (MIP) using all or selected slices, including single slices where this allowed optimal visualisation of the data. Whole mount image datasets of SRA and MRA were exported from Zeiss Zen Pro to IMARIS x64 (Bitplane, version 8.0.2, build 36053) for 3D and 3D stereo visualisation using the Surpass 3D view. Rendering in 3D was done using transparent (MIP) 3D mode and sections performed with the OrthoSlicer and Clipping tools. Single and multi-channel images were exported after optimisation of brightness and contrast in TIFF file format. Videos were made using the inbuilt IMARIS point-to-point animation tool and then exported directly in MP4 file format. For Iodinated microfocus computed tomography, projection images were reconstructed with modified Feldkamp filtered back-projection algorithms with proprietary software (CTPro3D, Nikon Metrology) and post processed with VGStudio MAX (Volume Graphics GmbH, Version 3.2).

RNA sequencing analysis: The identified genes were filtered out if not expressed at a count-per-million (CPM) higher than 1 in at least 2 samples of at least one condition. Data were normalized using the trimmed mean of M-values normalization method (TMM) implemented in edgeR package (version 3.40.2) 43. A Principal Component Analysis (PCA) was performed by R function prcomp on log2(CPM+1) data, after centering. Data visualization was performed with R package ggplot2 v. 3.4.2 44. Differentially expressed genes (DEGs) were computed with edgeR, using a mixed criterion based on p-value, after false discovery rate (FDR) correction by Benjamini-Hochberg method, lower than 0.01 and absolute fold change higher than 2, unless otherwise specified. Hierarchical clustering and heat map visualization were performed using R package pheatmap v.1.0.12 with Euclidean distance and complete linkage. Functional enrichment analysis of Reactome pathways (updated in 2022) was performed by right-sided hypergeometric test using ClueGO (version 2.5.10) 45 and CluePedia (version 1.5.10) 46 within Cytoscape v. 3.10.0 environment 47. Proteins playing a role as ligands or receptors were selected according to Ramilowski et al. 48
Receptors identified as DEGs in the comparison of MRA vs. organoids or SRA in a specific subtissue, were used to subselect the corresponding ligands. The receptor-ligand pairs were further shortlisted by including only ligands that were DEGs and almost not expressed in the subtissue of interest in organoids or SRA.

Thiol organoid barcoding in situ mass cytometry (TOBis MC) analysis: Raw data was processed using StandardBioTools CyTOF software v.6.7 to remove EQ beads and normalise signals prior to exporting data as FCS files. After acquisition, the processed file was debarcoded (https://github.com/zunderlab/single-cell-debarcoder) and uploaded onto the Cytobank platform for further analysis. Single cells were gated for Gaussian parameters, Cisplatinlow and DNAhigh to remove debris, doublets, and any other non-cellular resembling matter from the data. UMAP analysis was performed using the default settings in Cytobank (https://www.cytobank.org/). The individual debarcoded files were downloaded from Cytobank as text files and using the Mass Cytometry data analysis pipeline, CyGNAL (https://github.com/TAPE-Lab/CyGNAL), they were pre-processed and the Earth Mover's Distance (EMD) scores were computed. Once computed, heatmaps and PCA plots were generated and compiled using OmniGraffle professional.

WES (Simple Western™ Automated Western Blot Systems): Results were visualized using the Compass for Simple Western Software v.6.1.0.

qRT-PCR results were analysed with QuantStudio Design & Analysis Software (v1.5.2, Thermo Fisher). GAPDH expression was used to normalize Ct values for gene expression, and data were shown as relative fold change to controls (Antrum organoids), using ��Ct method, and presented as mean � SEM.

GraphPad Prism Mac (v.10.3.0) was used for graphs and statistical analysis.

For manuscripts utilizing custom algorithms or software that are central to the research but not yet described in published literature, software must be made available to editors and reviewers. We strongly encourage code deposition in a community repository (e.g. GitHub). See the Nature Portfolio guidelines for submitting code & software for further information.

## Data

Policy information about availability of data

All manuscripts must include a data availability statement. This statement should provide the following information, where applicable:

- Accession codes, unique identifiers, or web links for publicly available datasets
- A description of any restrictions on data availability
- For clinical datasets or third party data, please ensure that the statement adheres to our policy

The authors declare that all data supporting the findings of this study are available within the article, its Supplementary Information, Source Data files, and online deposited data. The Gastric RNA-seq data generated in this study have been deposited in the NCBI GEO database and are publicly available at the records GSE247115 and GSE280843. Reactome pathways Database (https://reactome.org) has been used for Functional Enrichment Analysis. The thiol organoid barcoding in situ mass cytometry (TOBis MC) data are deposited on https://community.cytobank.org/cytobank/experiments/113293.

## Research involving human participants, their data, or biological material

Policy information about studies with human participants or human data. See also policy information about sex, gender (identity/presentation), and sexual orientation and race, ethnicity and racism.

Reporting on sex and gender

The sex of the specimens was not considered as a parameter for the study design. Both male and female samples were included in the single cell RNA sequencing or used for organoids derivation and analysis.

Reporting on race, ethnicity, or

Race or ethnicity were not considered as a parameter for the study design.

| | |
|---|---|
| Reporting on race, ethnicity, or other socially relevant | |
| Population characteristics | 4yo female<br>7yo male<br>6mo female<br>11yo male<br>8yo male<br>8yo male<br>14yo male<br>16yo male |
| Recruitment | Human fetal stomachs were dissected from tissue obtained immediately after termination of pregnancy, in compliance with the bioethics legislation in the UK.<br><br>Human pediatric gastric surgical biopsies were collected after patient informed consent, in compliance with all relevant ethical regulations for work with human participants. All pediatric gastric tissue was donated by the patient, or person with parental responsibility.<br><br>Informed consent was undertaken by an independent research coordinator from the BRC.  The authors were not involved in any phase of the consent process and every patient (or their parent or person with parental responsibility, for minors) undergoing gastric surgery or upper gastrointestinal endoscopy at Great Ormond Street Hospital was approached by the research coordinator for involvement in the study. |
| Ethics oversight | Human fetal stomachs were dissected from tissue obtained immediately after termination of pregnancy, in compliance with the bioethics legislation in the UK. Fetal samples were obtained from the Joint MRC/Wellcome Trust Human Developmental Biology Resource (HDBR) under Research Tissue Bank ethics approval UCL site REC reference: 18/LO/0822 - IRAS project ID: 244325. Samples were used for tissue characterisation.<br><br>All paediatric gastric tissue was donated by the patient, or person with parental responsibility, after informed consent was obtained by an independent research coordinator at the relevant study site, under the approved research license. Human gastric biopsies were collected and processed in compliance with the Human Tissue Act under licence 18DS02 by the NHS Health Research Authority, East of England, Cambridge Central Research Ethics Committee. Paediatric samples were sourced from endoscopic mucosal biopsies at Great Ormond Street Hospital NHS Foundation Trust. Samples were used for deriving organoid lines or for native tissue characterisation. |

Note that full information on the approval of the study protocol must also be provided in the manuscript.

# Field-specific reporting

Please select the one below that is the best fit for your research. If you are not sure, read the appropriate sections before making your selection.

☒ Life sciences ☐ Behavioural & social sciences ☐ Ecological, evolutionary & environmental sciences

For a reference copy of the document with all sections, see nature.com/documents/nr-reporting-summary-flat.pdf

# Life sciences study design

All studies must disclose on these points even when the disclosure is negative.

| | |
|---|---|
| Sample size | Sample size for every experiment performed and image acquired is presented in the figure legends or in the Method section.<br><br>For imaging data, images in the manuscript are representative images of a minimum n=3 experiments, with sample size determined by the availability of patient samples. For this study,  a minimum of 3 healthy patients lines were used for each experiment. For affected patients,  4 PMM2 patient lines were used for evaluation of organoid dimension and proliferation; 2 patient lines were used for protein and functional analysis.<br><br>For quantitative analyses, no sample size calculation was performed but the sample size / replicate number was chosen in order to provide sufficient data points for the determination of measures of central tendency, variance, and parametric vs non-parametric distribution of the data.<br><br>Statistical significance of reported results was assessed by statistical tests during data analyses as indicated in Figure Legends. |
| Data exclusions | No data were excluded from the analyses. |
| Replication | Tissue samples were stained from 2 different patients, by 2 independent operators (1b, Extended data 1a-b). Cell culture experiments were replicated within our laboratory by at least three independent operators, with a minimum of n=3 times per experiment. Assembloid formation protocol was optimized by four different operators, using >10 organoids and collagen concentrations. Final optimized settings were used to |

performe all following experiments in the manuscript (Extended data 2). Immunofluorescence stainings of organoids/MRAs/Tissue/MRA slices were performed >3 times by two independent operators (1f, 2b-I, 4d, Extended data 2e,f, 8a,b). Western Simple analyses were performed n=3 times by one operator. Functional assays (acridine orange) were repeated n=3 times by one operator, with blind analysis performed by another operator (4c, 5k, Extended data 8c). Hyperplastic-like budding formations were assessed n=3 by immunostaining, n=1 by H&E (5e,f). Mucin organoid staining was performed n=4 (5h). GFP/RFP organoid line labelling was performed once (Extended data 6). Mouse grafting experiments were repeated twice by two different operators. Sequencing analysis was performed once on each sample as reported in the Methods. TOBis analysis was performed once after a first pilot experimental setup with 3 technical replicates per condition.

All attempts at replication were successful.

| | |
|---|---|
| Randomization | Randomization was not required in this study because the study design intentionally compared experimental groups, organoids vs assembloids . This in vitro study includes appropriate positive and negative controls, as presented in the manuscript. |
| Blinding | Organoids diameters were analysed in blind and with same settings for controls and samples. The investigator analyzing the experiment was not the same investigator performing the experiment for all immunofluorescence and qPCR data. RNAseq analysis was done by the bioinfomatician independent of the laboratory investigators, who received the data already blinded to conceal their group allocations. Blinding was performed in a first stage of the data analysis, then it was not possible due to participants involvement in data discussion. |

# Reporting for specific materials, systems and methods

We require information from authors about some types of materials, experimental systems and methods used in many studies. Here, indicate whether each material, system or method listed is relevant to your study. If you are not sure if a list item applies to your research, read the appropriate section before selecting a response.

## Materials & experimental systems

| n/a | Involved in the study | |
|---|---|---|
| ☐ | ☒ | Antibodies |
| ☐ | ☒ | Eukaryotic cell lines |
| ☒ | ☐ | Palaeontology and archaeology |
| ☐ | ☒ | Animals and other organisms |
| ☒ | ☐ | Clinical data |
| ☒ | ☐ | Dual use research of concern |
| ☒ | ☐ | Plants |

## Methods

| n/a | Involved in the study | |
|---|---|---|
| ☒ | ☐ | ChIP-seq |
| ☒ | ☐ | Flow cytometry |
| ☒ | ☐ | MRI-based neuroimaging |

# Antibodies

Antibodies used

Antibody, Supplier, Catalog Number, Clone Name, Lot Number, Host, Dilution

ATPase H+/K+ transporting subunit beta (ATP4B), ThermoFisher, MA3-923, 2G11, YB370950, Mouse, 1:100
Chromogranin A (CHGA), Abcam, ab15160, poly, GR3190950-1, Rabbit, 1:50
E-cadherin (E-CAD), BD Bioscinece, 610181, Clone 36/E-Cadherin, 1158439, Mouse, 1:100
Gastrin (GAST), Abcam, ab223501, SP318, Gr3221209-3, Rabbit,1:100
Ghrelin (GHRL), Novus Biology, NB600-813, poly, P1, Goat, 1:100
Integrin beta-4 (INTB4), Abcam, ab110167, 439-9B, GR3385737-1, Rat, 1:200
Iroquois Homeobox 3 (IRX3), Santa Cruz, Sc-166657, F-8, K3022, Mouse, 1:100
Lysozyme (LYZ), GeneTex, GTX72913, polyclonal, 821604003, Rabbit, 1:100
Mucin 5AC (MUC5AC), Invitrogen, ma5 Mucin -12178, 45M1, YH383543, Mouse, 1:100
Mucin 6 (MUC6), Abcam, ab216017, MUC6/916, GR310618-9, Mouse, 1:100
Pancreatic and duodenal homeobox 1 (PDX1), R&D SYSTEMS, AF2419, polyclonal, UNY0120041, Goat, 1:100
Pepsinogen (PGC), Atlas, HPA031717, polyclonal, A106241, Rabbit, 1:100,
Somatostatin (SST), R&D Systems, MAB2358, 906552, CIVM0319071, Rat, 1:100

anti-Mouse 488 (secondary AB), Thermo Fisher, A11001, 1810918, Goat, 1:300
anti-Mouse 568 (secondary AB), Thermo Fisher, A10037, 1696197, Goat, 1:300
anti-Mouse 647 (secondary AB),Thermo Fisher, A31571 2720365, Donkey, 1:300
anti-Rabbit 568 (secondary AB), Thermo Fisher, A11042, 2743014, Donkey, 1:300
Anti-Rat 488 (secondary AB), Thermo Fisher, A48269, 2180272, Donkey, 1:300
Anti-Rabbit 647 (secondary AB), Thermo Fisher, A-21244, 1246457, Goat,1:300
Anti-Goat 633 (secondary AB), Thermo Fisher, A-21082, 2095396, Donkey, 1:300
anti-Rat 594 (secondary AB), Thermo Fisher, A11007, 2633534, Goat,1:300
Phalloidin 647, Thermo Fisher, A22287, n/a, 2015553, n/a, 1:500
Hoechst 33342 (nuclear staining), Thermo Fisher, H1399, n/a, 1932847, n/a, 10 µg/mL

TOBis antibodies: Antibody, Supplier, Catalog Number, Clone Name, Web site reerence, Host
RFP, eBiosciences, #200-301-379, 8E5.G7, https://www.thermofisher.com/antibody/product/RFP-Antibody-clone-8E5-G7-Monoclonal/200-301-379, Mouse
mCherry, Thero Fisher, M11217, 16D7, https://www.thermofisher.com/antibody/product/mCherry-Antibody-clone-16D7-Monoclonal/M11217#References, Rat
IdU, Fluidigm, Q-27194, https://store.standardbio.com/product_detail/guest-catalog/201127/

cCaspase3 [D175], CST, #9661, D3E3, https://www.cellsignal.com/products/primary-antibodies/cleaved-caspase-3-asp175-antibody/9661#citations, Rabbit
Stathmin1, Bio-techne, NBP1-76798, polyclonal, https://www.novusbio.com/products/stathmin-1-antibody_nbp1-76798#reviews-publications, Rabbit
pNDRG1 [T346] , CST, #5482, D98G11, https://www.cellsignal.com/products/primary-antibodies/phospho-ndrg1-thr346-d98g11-xp-rabbit-mab/5482#citations, Rabbit
Lysozyme, Biorad, 5490-4110, SB1 (BGN/06/961), https://www.bio-rad-antibodies.com/monoclonal/human-lysozyme-antibody-sb1-bgn-06-961-5790-4110.html?f=purified, Mouse
pSRC [Y418], eBioscience, 50-9034-42, SC1T2M3, https://www.thermofisher.com/antibody/product/Phospho-SRC-Tyr418-Antibody-clone-SC1T2M3-Monoclonal/50-9034-42#References, Mouse
p4E-BP1 [T37/46 ], CST, #2855, 236B4, https://www.cellsignal.com/products/primary-antibodies/phospho-4e-bp1-thr37-46-236b4-rabbit-mab/2855#citations, Rabbit
pRB [S807/811], BD Biosciences, 558389, J112-906, https://www.bdbiosciences.com/en-us/products/reagents/western-blotting-and-molecular-reagents/western-blot-reagents/purified-mouse-anti-rb-ps807-ps811.558389#citations&references, Mouse
pAKT [T308], BD Biosciences, 558275, J1-223.371, https://www.bdbiosciences.com/en-gb/products/reagents/flow-cytometry-reagents/research-reagents/single-color-antibodies-ruo/pe-mouse-anti-akt-pt308.558275#citations&references, Mouse
pCREB [S133], CST, #9198, 87G3, https://www.cellsignal.com/products/primary-antibodies/phospho-creb-ser133-87g3-rabbit-mab/9198#citations, Rabbit
Sox9, abcam, Ab76997, 3C10, https://www.abcam.com/products/primary-antibodies/sox9-antibody-3c10-bsa-and-azide-free-ab76997.html#references, Mouse
pMKK3/MKK6 [S189/207], CST, #9231, D8E9, https://www.cellsignal.com/products/primary-antibodies/phospho-mkk3-ser189-mkk6-ser207-antibody/9231#citations, Rabbit
pP38 [T180/Y182], CST, #9211, D3F9, https://www.cellsignal.com/products/primary-antibodies/phospho-p38-mapk-thr180-tyr182-antibody/9211#citations, Rabbit
Ghrelin1, Novus Biologicals, H00051738-M01, 2F4, https://www.novusbio.com/products/ghrelin-obestatin-antibody-2f4_h00051738-m01#reviews-publications, Mouse
Gastrin, Abcam, ab223501, SP318, https://www.abcam.com/products/primary-antibodies/gastrin-antibody-sp318-ab223501.html, Rabbit
LRIG1, R&D Systems, AF7498, polyclonal, https://www.rndsystems.com/products/human-lrig1-antibody_af7498#product-citations, Sheep
Pepsinogen C, Abcam, ab255826, EPR22843-269, https://www.abcam.com/products/primary-antibodies/pepsinogen-iipgc-antibody-epr22843-269-ab255826.html#references, Rabbit
pP120-Catenin [T310], BD Biosciences, 558203, 22/p120 (pT310), https://www.bdbiosciences.com/content/dam/bdb/products/global/reagents/western-blotting-and-molecular-reagents/western-blot-reagents/558xxx/5582xx/558203_base/pdf/558203.pdf, Mouse
MUC5AC, Invitrogen, MA5-12178, 45M1, https://www.thermofisher.com/antibody/product/MUC5AC-Antibody-clone-45M1-Monoclonal/MA5-12178#references, Mouse
pGSK3b [S9], CST, #9336, D85E12, https://www.cellsignal.com/products/primary-antibodies/phospho-gsk-3b-ser9-antibody/9336#citations, Rabbit
Mucin 6 (MUC6), Abcam, ab216017, MUC6/916, https://www.abcam.com/products/primary-antibodies/gastric-mucinmuc-6-antibody-muc6916-ab216017.html#references, Mouse
pSMAD2 [S465/467] /pSMAD3 [S423/425], CST, #8828, D27F4, https://www.cellsignal.com/products/primary-antibodies/phospho-smad2-ser465-467-smad3-ser423-425-d27f4-rabbit-mab/8828#citations, Rabbit
CHGA, Santa Cruz, sc-393941, C-12, https://www.scbt.com/p/chr-a-antibody-c-12#product_citations, Dil, Mouse
Somatostatin, Atlas, MAB2358, 906552, https://www.rndsystems.com/products/human-mouse-somatostatin-antibody-906552_mab2358#citations, Rat
cPARP [D214], CST, #5626, D64E10, https://www.cellsignal.com/products/primary-antibodies/cleaved-parp-asp214-d64e10-xp-rabbit-mab/5625#citations, Rabbit
K-ATPase_ATP4B, Abcam, ab176992, polyclonal, https://www.abcam.com/products/primary-antibodies/hydrogen-potassium-atpase-betaatp4b-antibody-ab176992.html#references, Mouse
Cyclin B1, BD Biosciences, 554179, GNS-11, https://www.bdbiosciences.com/en-eu/products/reagents/flow-cytometry-reagents/research-reagents/single-color-antibodies-ruo/purified-mouse-anti-cyclin-b1.554179#citatioons&references, Mouse, Dil
pHistone H3, BioLegens, 641010, [S28] HTA28, https://www.biolegend.com/en-gb/products/pe-anti-histone-h3-phosphorylated-ser28-antibody-11862?GroupID=GROUP26, Rat
Pancreatic and duodenal homeobox 1 (PDX1), R&D SYSTEMS, AF2419, polyclonal, https://www.rndsystems.com/products/human-pdx-1-ipf1-antibody_af2419#product-citations, Goat
GFP, eBiosciences, 50-6498-82, F512.4, https://www.thermofisher.com/antibody/product/GFP-Antibody-clone-5F12-4-Monoclonal/50-6498-82#references, Rat
LGR5, R&D Systems, MAB82401, 889901, https://www.novusbio.com/products/lgr5-gpr49-antibody-889901_mab82401#reviews-publications, Rat

Validation

Antibodies were validated on primary gastric tissue and using validation information on the manufacturer's website. Links to validation information from the manufacturers are included here:

ATPase H+/K+ transporting subunit beta (ATP4B), ThermoFisher, MA3-923, https://www.thermofisher.com/antibody/product/ATP4B-Antibody-clone-2G11-Monoclonal/MA3-923
Chromogranin A (CHGA), Abcam, ab15160, https://www.abcam.com/chromogranin-a-antibody-ab15160.html
E-cadherin (E-CAD), BD Bioscinece, 610181, https://www.bdbiosciences.com/en-gb/products/reagents/microscopy-imaging-reagents/immunofluorescence-reagents/purified-mouse-anti-e-cadherin.610181
Gastrin (GAST), Abcam, ab223501, https://www.abcam.com/products/primary-antibodies/gastrin-antibody-sp318-ab223501.html
Ghrelin (GHRL), Novus Biology, NB600-813, https://www.novusbio.com/products/ghrelin-obestatin-antibody_nb600-813
Integrin beta-4 (INTB4), Abcam, ab110167, https://www.abcam.com/integrin-beta-4-antibody-439-9b-ab110167.html
Iroquois Homeobox 3 (IRX3), Santa Cruz, Sc-166657, https://www.scbt.com/p/irx3-antibody-f-8
Lysozyme (LYZ), GeneTex, GTX72913, https://www.genetex.com/Product/Detail/Lysozyme-antibody/GTX72913
Mucin 5AC (MUC5AC), Invitrogen, ma5 Mucin -12178, https://www.thermofisher.com/antibody/product/MUC5AC-Antibody-

clone-45M1-Monoclonal/MA5-12178
Mucin 6 (MUC6), Abcam, ab216017, https://www.abcam.com/gastric-mucinmuc-6-antibody-muc6916-ab216017.html
Pancreatic and duodenal homeobox 1 (PDX1), R&D SYSTEMS, AF2419, https://www.rndsystems.com/products/human-pdx-1-ipf1-antibody_af2419
Pepsinogen (PGC), Atlas, HPA031717, https://www.atlasantibodies.com/products/antibodies/primary-antibodies/triple-a-polyclonals/pgc-antibody-hpa031717/
Somatostatin (SST), R&D Systems, MAB235, https://www.rndsystems.com/products/human-mouse-somatostatin-antibody-906552_mab2358

anti-Mouse 488 (secondary AB), Thermo Fisher, A11001, https://www.thermofisher.com/antibody/product/Goat-anti-Mouse-IgG-H-L-Cross-Adsorbed-Secondary-Antibody-Polyclonal/A-11001
anti-Mouse 568 (secondary AB), Thermo Fisher, A10037, https://www.thermofisher.com/antibody/product/Donkey-anti-Mouse-IgG-H-L-Highly-Cross-Adsorbed-Secondary-Antibody-Polyclonal/A10037
anti-Mouse 647 (secondary AB),Thermo Fisher, A31571, https://www.thermofisher.com/antibody/product/Donkey-anti-Mouse-IgG-H-L-Highly-Cross-Adsorbed-Secondary-Antibody-Polyclonal/A-31571
anti-Rabbit 568 (secondary AB), Thermo Fisher, A11042, https://www.thermofisher.com/antibody/product/A10042.html?ef_id=Cj0KCQjw4eaJBhDMARIsANhrQAAUQ-
Anti-Rat 488 (secondary AB), Thermo Fisher, A48269, https://www.thermofisher.com/antibody/product/Donkey-anti-Rat-IgG-H-L-Highly-Cross-Adsorbed-Secondary-Antibody-Polyclonal/A48269
Anti-Rabbit 647 (secondary AB), Thermo Fisher, A-21244, https://www.thermofisher.com/antibody/product/Goat-anti-Rabbit-IgG-H-L-Cross-Adsorbed-Secondary-Antibody-Polyclonal/A-21244
Anti-Goat 633 (secondary AB), Thermo Fisher, A-21082, https://www.thermofisher.com/antibody/product/Donkey-anti-Goat-IgG-H-L-Cross-Adsorbed-Secondary-Antibody-Polyclonal/A-21082
anti-Rat 594 (secondary AB), Thermo Fisher, A11007, https://www.thermofisher.com/antibody/product/Goat-anti-Rat-IgG-H-L-Cross-Adsorbed-Secondary-Antibody-Polyclonal/A-11007
Phalloidin 647, Thermo Fisher, A22287, https://www.thermofisher.com/order/catalog/product/A22287#/A22287
Hoechst 33342 (nuclear staining), Thermo Fisher, H1399, https://www.thermofisher.com/order/catalog/product/H1399#/H1399

Subsequent staining protocols included positive and negative controls.

TOBis antibodies:
RFP, eBiosciences, #200-301-379, 8E5.G7, https://www.thermofisher.com/antibody/product/RFP-Antibody-clone-8E5-G7-Monoclonal/200-301-379
mCherry, Thero Fisher, M11217, 16D7, https://www.thermofisher.com/antibody/product/mCherry-Antibody-clone-16D7-Monoclonal/M11217
IdU, Fluidigm, Q-27194, https://store.standardbio.com/product_detail/guest-catalog/201127/
cCaspase3 [D175], CST, #9661, D3E3, https://www.cellsignal.com/products/primary-antibodies/cleaved-caspase-3-asp175-antibody/9661
Stathmin1, Bio-techne, NBP1-76798, polyclonal, https://www.novusbio.com/products/stathmin-1-antibody_nbp1-76798#
pNDRG1 [T346] , CST, #5482, D98G11, https://www.cellsignal.com/products/primary-antibodies/phospho-ndrg1-thr346-d98g11-xp-rabbit-mab/5482
Lysozyme, Biorad, 5490-4110, SB1 (BGN/06/961), https://www.bio-rad-antibodies.com/monoclonal/human-lysozyme-antibody-sb1-bgn-06-961-5790-4110.html?f=purified
pSRC [Y418], eBioscience, 50-9034-42, SC1T2M3, https://www.thermofisher.com/antibody/product/Phospho-SRC-Tyr418-Antibody-clone-SC1T2M3-Monoclonal/50-9034-42
p4E-BP1 [T37/46 ], CST, #2855, 236B4, https://www.cellsignal.com/products/primary-antibodies/phospho-4e-bp1-thr37-46-236b4-rabbit-mab/2855
pRB [S807/811], BD Biosciences, 558389, J112-906, https://www.bdbiosciences.com/en-us/products/reagents/western-blotting-and-molecular-reagents/western-blot-reagents/purified-mouse-anti-rb-ps807-ps811.558389
pAKT [T308], BD Biosciences, 558275, J1-223.371, https://www.bdbiosciences.com/en-gb/products/reagents/flow-cytometry-reagents/research-reagents/single-color-antibodies-ruo/pe-mouse-anti-akt-pt308.558275
pCREB [S133], CST, #9198, 87G3, https://www.cellsignal.com/products/primary-antibodies/phospho-creb-ser133-87g3-rabbit-mab/9198
Sox9, abcam, Ab76997, 3C10, https://www.abcam.com/products/primary-antibodies/sox9-antibody-3c10-bsa-and-azide-free-ab76997.html
pMKK3/MKK6 [S189/207], CST, #9231, D8E9, https://www.cellsignal.com/products/primary-antibodies/phospho-mkk3-ser189-mkk6-ser207-antibody/9231
pP38 [T180/Y182], CST, #9211, D3F9, https://www.cellsignal.com/products/primary-antibodies/phospho-p38-mapk-thr180-tyr182-antibody/9211
Ghrelin1, Novus Biologicals, H00051738-M01, 2F4, https://www.novusbio.com/products/ghrelin-obestatin-antibody-2f4_h00051738-m01
Gastrin, Abcam, ab223501, SP318, https://www.abcam.com/products/primary-antibodies/gastrin-antibody-sp318-ab223501.html
LRIG1, R&D Systems, AF7498, polyclonal, https://www.rndsystems.com/products/human-lrig1-antibody_af7498
Pepsinogen C, Abcam, ab255826, EPR22843-269, https://www.abcam.com/products/primary-antibodies/pepsinogen-iipgc-antibody-epr22843-269-ab255826.html
pP120-Catenin [T310], BD Biosciences, 558203, 22/p120 (pT310), https://www.bdbiosciences.com/content/dam/bdb/products/global/reagents/western-blotting-and-molecular-reagents/western-blot-reagents/558xxx/5582xx/558203_base/pdf/558203.pdf
MUC5AC, Invitrogen, MA5-12178, 45M1, https://www.thermofisher.com/antibody/product/MUC5AC-Antibody-clone-45M1-Monoclonal/MA5-12178
pGSK3b [S9], CST, #9336, D85E12, https://www.cellsignal.com/products/primary-antibodies/phospho-gsk-3b-ser9-antibody/9336
Mucin 6 (MUC6), Abcam, ab216017, MUC6/916, https://www.abcam.com/products/primary-antibodies/gastric-mucinmuc-6-antibody-muc6916-ab216017.html
pSMAD2 [S465/467] /pSMAD3 [S423/425], CST, #8828, D27F4, https://www.cellsignal.com/products/primary-antibodies/phospho-smad2-ser465-467-smad3-ser423-425-d27f4-rabbit-mab/8828
CHGA, Santa Cruz, sc-393941, C-12, https://www.scbt.com/p/chr-a-antibody-c-12#product_citations, Dil, Mouse
Somatostatin, Atlas, MAB2358, 906552, https://www.rndsystems.com/products/human-mouse-somatostatin-

antibody-906552_mab2358
cPARP [D214], CST, #5626, D64E10, https://www.cellsignal.com/products/primary-antibodies/cleaved-parp-asp214-d64e10-xp-rabbit-mab/5625
K-ATPase_ATP4B, Abcam, ab176992, polyclonal, https://www.abcam.com/products/primary-antibodies/hydrogen-potassium-atpase-betaatp4b-antibody-ab176992.html
Cyclin B1, BD Biosciences, 554179, GNS-11, https://www.bdbiosciences.com/en-eu/products/reagents/flow-cytometry-reagents/research-reagents/single-color-antibodies-ruo/purified-mouse-anti-cyclin-b1.554179#citatioons&references, Mouse, Dil
pHistone H3, BioLegens, 641010, [S28] HTA28, https://www.biolegend.com/en-gb/products/pe-anti-histone-h3-phosphorylated-ser28-antibody-11862?GroupID=GROUP26
Pancreatic and duodenal homeobox 1 (PDX1), R&D SYSTEMS, AF2419, polyclonal, https://www.rndsystems.com/products/human-pdx-1-ipf1-antibody_af2419
GFP, eBiosciences, 50-6498-82, F512.4, https://www.thermofisher.com/antibody/product/GFP-Antibody-clone-5F12-4-Monoclonal/50-6498-82
LGR5, R&D Systems, MAB82401, 889901, https://www.novusbio.com/products/lgr5-gpr49-antibody-889901_mab82401

## Eukaryotic cell lines

Policy information about cell lines and Sex and Gender in Research

Cell line source(s): Human pediatric cells were isolated from gastric surgical biopsies and endoscopies immediately after sample collection.

Authentication: Organoid lines were not authenticated.

Mycoplasma contamination: All cell lines used in this work were tested monthly and always tested negative for Mycoplasma.

Commonly misidentified lines (See ICLAC register): No commonly misidentified cell lines were used in the study

## Animals and other research organisms

Policy information about studies involving animals; ARRIVE guidelines recommended for reporting animal research, and Sex and Gender in Research

Laboratory animals: 12 week old non-obese diabetic severe combined immunodeficient interleukin 2 receptor gamma null (NOD scid gamma, NSG, NOD.Cg-Prkdcscid Il2rgtm1Wjl/SzJ) mice from Charles River Laboratories were used in all in vivo implantation experiments. Mice were group housed in individually ventilated cages in a specialised immunodeficient colony room, in in 12-hour light-dark cycles at 20-24°C, humidity 45-65%, with food and water ad libitum. Following subcutaneous implantation procedures, mice were recovered individually in new individually ventilated cages, before being returned to group housing with other mice who had undergone the same procedure. Access to standard diet and water was immediately provided following recovery. Subcutaneous pockets were created by blunt dissection according to the number of samples being implanted (maximum 4 per mouse).

Wild animals: No wild animals were used in the study

Reporting on sex: Female mice were used for all subcutaneous implants.

Field-collected samples: No field collected samples were used in the study

Ethics oversight: All animal care and procedures were performed in accordance with National Centre for the Replacement Refinement and Reduction of Animals in Research (NC3Rs) guidelines.
Experimental work was undertaken under ethics approval governed by UK Home Office Project Licence PPL PDD3A088A and Personal Licence PIL I73E168C9.

Note that full information on the approval of the study protocol must also be provided in the manuscript.

## Plants

Seed stocks: Not applicable

Novel plant genotypes: Not applicable

Authentication: Not applicable
